# LncRNAs serve as novel biomarkers for diagnosis and prognosis of childhood ALL

**DOI:** 10.1186/s40364-021-00303-x

**Published:** 2021-06-10

**Authors:** Xuanmei Huang, Libin Huang, Qing Xie, Ling Zhang, Shaohui Huang, Mingye Hong, Jiangbin Li, Zunnan Huang, Hua Zhang

**Affiliations:** 1 Institute of Laboratory Medicine, Guangdong Provincial Key Laboratory of Medical Molecular Diagnostics, Key Laboratory of Big Data Mining and Precision Drug Design, School of Medical Technology, Guangdong Medical University, Guangdong Medical University, 523808 Dongguan, China; 2 Department of Pediatrics, The First Affiliated Hospital, Sun Yat-sen University, 58 Zhong shan Er Lu, 510080 Guangzhou, China; 3 Health Science Center, The University of Texas, 77030 Houston, USA

**Keywords:** LncRNAs, Childhood acute lymphoblastic leukemia, Leukemia-free survival, Proliferation, Apoptosis

## Abstract

**Background:**

Although some studies have demonstrated that lncRNAs are dysregulated in hematopoietic malignancies and may regulate the progression of leukemia, the detailed mechanism underlying tumorigenesis is still unclear. This study aimed to investigate lncRNAs that are differentially expressed in childhood B-cell acute lymphoblastic leukemia (B-ALL) and T-cell acute lymphoblastic leukemia (T-ALL) and their potential roles in the progression of childhood ALL.

**Methods:**

Microarrays were used to detect differentially expressed lncRNAs and mRNAs. Several aberrantly expressed lncRNAs were validated by qRT-PCR. Leukemia-free survival was analyzed using the Kaplan–Meier method with a log-rank test. The co-expression correlations of lncRNAs and mRNAs were determined by Spearman’s correlation coefficient. CCK-8 assays and flow cytometry were performed to measure cell proliferation and apoptosis.

**Results:**

We revealed that many lncRNAs were abnormally expressed in B-ALL and T-ALL. LncRNA/mRNA co-expression and the gene locus network showed that dysregulated lncRNAs are involved in diverse cellular processes. We also assessed the diagnostic value of the differentially expressed lncRNAs and confirmed the optimal combination of TCONS_00026679, uc002ubt.1, ENST00000411904, and ENST00000547644 with an area under the curve of 0.9686 [95 % CI: 0.9369–1.000, *P* < 0.001], with 90.7 % sensitivity and 92.19 % specificity, at a cut-off point of -0.5700 to distinguish childhood B-ALL patients from T-ALL patients, implying that these specific lncRNAs may have potential to detect subsets of childhood ALL. Notably, we found that the 8-year leukemia-free survival of patients with high TCONS_00026679 (*p* = 0.0081), ENST00000522339 (*p* = 0.0484), ENST00000499583 (*p* = 0.0381), ENST00000457217 (*p* = 0.0464), and ENST00000451368 (*p* = 0.0298) expression levels was significantly higher than that of patients with low expression levels of these lncRNAs, while patients with high uc002ubt.1 (*p* = 0.0499) and ENST00000547644 (*p* = 0.0451) expression levels exhibited markedly shorter 8-year leukemia-free survival. In addition, some lncRNAs were found to play different roles in cell proliferation and apoptosis in T-ALL and B-ALL.

**Conclusions:**

Dysregulated lncRNAs involved in different regulatory mechanisms underlying the progression of childhood T-ALL and B-ALL might serve as novel biomarkers to distinguish ALL subsets and indicate poor outcomes.

**Supplementary Information:**

The online version contains supplementary material available at 10.1186/s40364-021-00303-x.

## Background

Acute lymphoblastic leukemia (ALL), the most common pediatric malignancy, is the leading cause of cancer-related death in children and is classified into B-cell acute lymphoblastic leukemia (B-ALL) and T-cell acute lymphoblastic leukemia (T-ALL) [1–3]. B-ALL and T-ALL can be further subdivided based on recurrent karyotypic abnormalities, including aneuploidy and translocations, which are usually associated with unfavorable clinical features and aggressive biological behavior [4–6]. Although recent adaptation of pediatric treatment regimens has dramatically improved the 5-year survival rate of patients with newly diagnosed childhood ALL to greater than 90 % in the developed world, unfortunately, approximately 15 % of children still experience refractory or relapsed ALL, which has a dismal prognosis [7–9]. Thus, it is urgent to identify novel biomarkers for early diagnosis and prognosis as well as new drug targets to enhance the overall outcomes for childhood ALL [10].

Long noncoding RNAs (lncRNAs) are greater than 200 nucleotides in length and play important roles in a wide range of biological processes, including tumorigenesis [11]. Many studies have demonstrated that aberrant lncRNAs can regulate gene expression at the transcriptional, posttranscriptional and translational levels, ultimately causing breast cancer, hepatocellular cancer, mixed-lineage leukemia, colorectal cancer, etc. [12–15]. In childhood leukemia, it has been reported that lnc-THADA-4, lnc-ACOT9-1 and lnc-NRIR may serve as novel therapeutic targets in myelomonocytic leukemia [16]. Lnc-CCDC26, lnc-DARS-AS1 and lnc-SNHG14 play oncogenic roles in childhood acute myeloid leukemia (AML) [17–19]. Moreover, a genome-wide lncRNA expression study revealed that some lncRNAs are involved in the progression of childhood MLL-rearranged ALL and might represent novel biomarkers [20]. It has also been found that lnc-INSR might promote immune suppression via enhancing Treg cell differentiation through the phosphatidylinositide 3-kinase/AKT signalling pathway in childhood ALL [21]. More recently, some lncRNAs, including novel lncRNAs, were found to be involved in the pathogenesis, prognosis and therapy of childhood ALL [20–25]. However, the detailed mechanism underlying childhood ALL is still largely unknown.

To identify differentially expressed lncRNA patterns and elucidate their potential roles in childhood T-ALL and B-ALL, microarray chips were used for the characterization of genome-wide lncRNAs and mRNAs in bone marrow samples, including 11 B-ALL, 11 T-ALL and 6 normal control samples. Some aberrant differentially expressed lncRNAs were confirmed in a validation cohort of 64 B-ALL, 43 for T-ALL and 21 healthy patients. Furthermore, the 8-year leukemia-free survival of patients with highly expressed lncRNAs, lncRNA/mRNA co-expression and gene locus networks was investigated. This study may provide a better understanding of the underlying mechanism and treatment of childhood ALL.

## Methods

### Patient samples and cell lines

Clinically, the immunophenotype of leukemia cells were defined by flow cytometry using full panel of markers for ALL in every newly diagnosed patient, as follows: TdT+, CD19+, CD79a+, CD10±, CD 20±, cyIgM±, surface immunoglobulin (sIg)- for B-ALL; and TdT+, cyCD3+, CD7 + for T-ALL [26, 27]. In this study, the inclusion and exclusion criteria are: the patients who firstly diagnosed as B-ALL or T-ALL were included, while the patients with ambiguous lineage or germ line predisposition were excluded. The healthy umbilical cord blood samples were used as the controls. All samples were obtained with informed consent from the First Affiliated Hospital of Sun Yat-sen University. Sample collection was approved by the Hospital’s Protection of Human Subjects Committee. Detailed information on the patients is summarized in Table 1. Bone marrow samples were obtained from 129 patients at the time of initial diagnosis, including 75 patients with B-ALL and 54 patients with T-ALL. 27 healthy umbilical cord blood samples have been also recruited. Among these samples, 11 B-ALL, 11 T-ALL and 6 healthy samples were used for the microarray, and 64 B-ALL, 43 T-ALL and 21 healthy samples were used for qRT-PCR validation. Jurkat and SUP-B15 ALL cell lines were purchased from American Type Culture Collection (ATCC, USA). All the cells were grown in RPMI-1640 medium (HyClone, USA) with 10 % fetal bovine serum (HyClone, USA) at 37 °C in a 5 % CO_2_ atmosphere.

**Table 1:** Characteristics of patients

Immunophenotype	T	B
**ALL (***N*** = 129)**	54	75
**Age at diagnosis, y (Mean (range))**	8.00 (0.92–13.60)	4.05 (0.17-14.00)
**Sex**		
Male	39	56
Female	15	19
**WBC count, × 10**^**9**^**/L (Mean (range))**	147.70 (2.00-632.50)	32.10 (1.700-557.6)
**FAB**		
L1	10	20
L2	34	38
L3	2	9
N/A	5	8
**Risk group**		
SR	4	9
MR	16	34
HR	28	24
N/A	6	8
**Cytogenetics**		
BCR/ABL positive	0	2
MLL positive	10	20
Both negative	34	53
**Healthy Control (***N*** = 27)**		

### LncRNA microarray profiling and qRT-PCR analysis

Samples were homogenized in liquid nitrogen. TRIzol reagent (Invitrogen, USA) was used to extract total RNA. The Arraystar Human LncRNA Array v2.0 platform (Kangcheng, China) was used to detect and normalize the data, and differentially expressed lncRNAs and mRNAs with significance passed volcano plot filtering (fold change > 2.0, *P* < 0.05, t-test). For qRT-PCR validation, total RNA was reverse transcribed into cDNA by the PrimeScript® RT Reagent Kit with gDNA Eraser (TaKaRa, Japan). Then, cDNA products were further quantified by SYBR Premix Ex TaqTM II (TaKaRa, China)-based qRT-PCR. All data were analysed in triplicate and normalized to the housekeeping gene GAPDH. All the primers are listed in Table S1.

### Co-expression networks and gene ontology analysis

The co-expression correlations of lncRNAs and mRNAs were determined by Spearman’s correlation coefficient. The Gene Ontology Consortium database (http://www.geneontology.org) was used to analyze the biological processes and molecular function categories [28].

### Cell transfection

Small interfering RNAs (siRNAs) against selected lncRNAs were obtained from GenePharma (GenePharma, China). The Neon Transfection System (Invitrogen, USA) was used for Jurkat and SUP-B15 cell transfection.

### Cell proliferation and apoptosis assays

Cell proliferation was measured using the Cell Counting Kit-8 (CCK-8). After siRNA transfection, 1 × 10^4^ cells/well were plated into 96-well plates, and CCK-8 assays (Dojindo Molecular Technologies, China) was performed after 0 h, 24 h, 48 h, 72 h, and 96 h. For the apoptosis assay, cells were transfected with siRNA, and 24 h later, a dexamethasone-induced killing agent was added. Finally, an Annexin V-PI Kit was used (Nanjing Keygen, China). A FACS Calibur with Cell Quest software (BDIS, USA) was used for flow cytometry and analysis.

### Statistical analysis

SPSS PASW Statistics (version 20.0) and GraphPad Prism (version 8.0) were used for statistical calculations. The Shapiro-Wilk test was used to identify whether the clinical data followed the normal distribution or not. For the clinical data analysis, Kruskal-Wallis test was implemented among three groups comparisons, and multiple comparisons were performed using one-way ANOVA and least significant difference t (LSD-t) test after the relative expression was ranked. The diagnostic utility of lncRNAs was determined by receiver operating characteristic (ROC) curves. Discriminant analysis was used to build a combination model of predicted probability, and the optimal cut-off point was the maximum Youden’s index. *P* < 0.05 was considered statistically significant. The probability of the leukemia-free survival at 8 years was the study endpoint. Leukemia-free survival was calculated from the date of complete remission (CR) until either death or relapse during remission. Analysis of leukemia-free survival was performed according to the Kaplan–Meier method. Two-tailed tests were used for univariate comparisons. A Cox proportional hazard regression model was used for univariate and multivariate analysis of prognostic factors.

## Results

### General expression profiles of differentially specific lncRNAs in childhood B-ALL and T-ALL

**Fig. 1:**
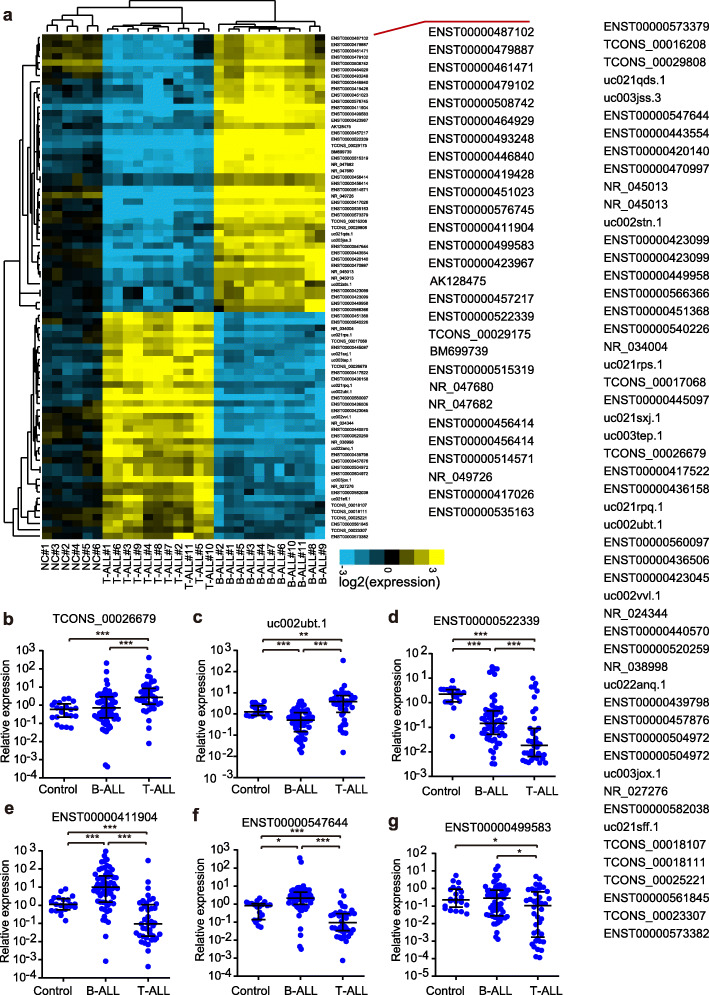
LncRNA profiles in childhood B-ALL and T-ALL patients. **A**. Cluster analysis of lncRNA expression in 11 childhood T-ALL patients, 11 B-ALL patients and 6 healthy controls (fold-change > 2.0). **B-G**. The aberrant expression profile of lncRNAs was validated by qPCR. Relative expression levels of lncRNAs were normalized to GAPDH

To explore the possible functions of the lncRNAs involved in the progression of B-ALL and T-ALL, we first performed a lncRNA microarray-based gene expression profile assay with a set of childhood patient samples, including 11 T-ALL, 11 B-ALL and 6 healthy control samples. In unsupervised hierarchical clustering analysis, the differentially expressed lncRNAs were used to generate a heat map, and they clearly self-segregated into T-ALL, B-ALL and control clusters (Fig. S1). By performing a SAM analysis, we found that a large number of lncRNAs exhibited observably higher or lower expression profiles in both T-ALL and B-ALL samples than in the negative control sample, which may imply that they have a potential function underlying leukemia progression. Notably, we also identified 2176 lncRNAs displaying a significant expression pattern between B-ALL and T-ALL, and a list of the top 80 differentially expressed lncRNAs is shown in Fig. 1A. This result may be consistent with the notion that lncRNA expression is specific in these different subtypes of ALL. To validate the microarray results, a total of 12 top differentially expressed lncRNAs between T-ALL and B-ALL were chosen for qRT-PCR in a validation sample set (64 for B-ALL, 43 for T-ALL and 21 healthy control samples). As a result, ten of the selected lncRNAs, except for NR_045112 and ENST00000560097, showed different expression patterns in these three subsets, which was highly consistent with that of the microarray (Fig. 1B-G, Fig. S2). Surprisingly, many lncRNAs can be used to distinguished B-ALL samples from T-ALL samples with high confidence (*p* < 0.001), for example, TCONS_00026679 (Fig. 1B), uc002ubt.1 (Fig. 1C), ENST00000522339 (Fig. 1D), ENST00000411904 (Fig. 1E), and ENST00000547644 (Fig. 1F).

### Evaluation of the diagnostic value of lncRNAs in childhood B-ALL and T-ALL patients

**Fig. 2:**
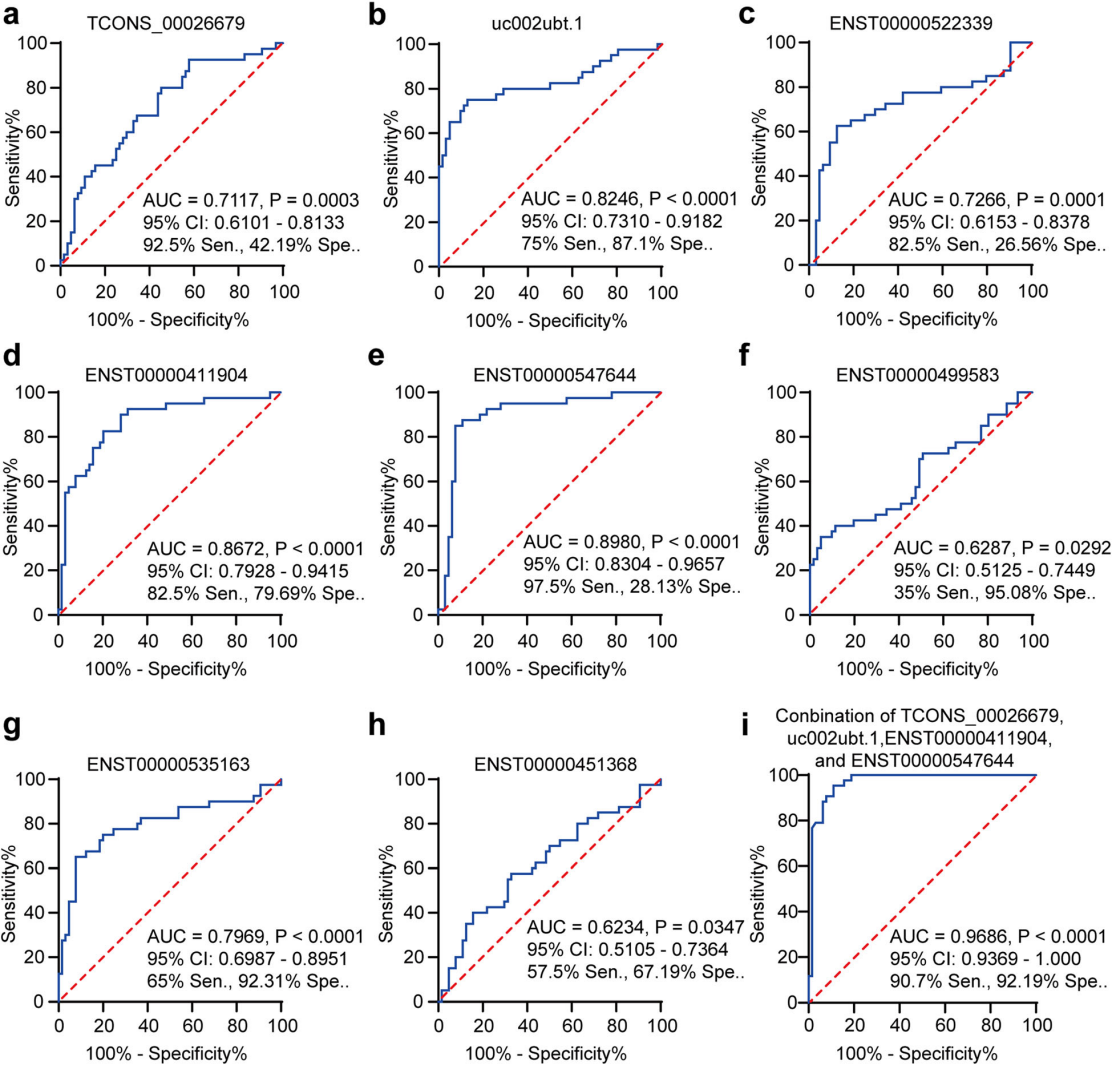
Diagnostic value of lncRNAs in childhood B-ALL and T-ALL patients. Diagnostic value of lncRNAs for childhood B-ALL and T-ALL patients: TCONS_00026679 (**A**), uc002ubt.1 (**B**), ENST00000522339 (**C**), ENST00000411904 (**D**), ENST00000547644 (**E**), ENST00000499583 (**F**), ENST00000535163 (**G**) and ENST00000451368 (**H**). **I**. The optimal combination of TCONS_00026679, uc002ubt.1, ENST00000411904, and ENST00000547644 to distinguish childhood B-ALL patients from T-ALL patients, and the AUC of PVP was up to 0.9686 [95 % CI: 0.9369–1.000, *P* < 0.001], with 90.7 % sensitivity and 92.19 % specificity, at a cut-off point of -0.5700

To analyze the diagnostic value of the selected lncRNAs in childhood B-ALL and T-ALL patients, ROC curve analysis was performed with log transformed relative expression of these lncRNAs. Subsequently, the associated area under the ROC curve (AUC) as well as the sensitivity (sen.) and specificity (spe.) were used to assess the diagnostic potential. We found that eight of the selected lncRNAs, not including ENST00000457217, NR_045112, ENST00000560097 and ENST00000436506, had a significant ability to distinguish childhood B-ALL from T-ALL patients (Fig. 2 and Fig. S3). As shown in Fig. 2, the AUC of ENST00000547644 was the highest, reaching 0.8980 [95 % CI: 0.8304–0.9657, *P* < 0.0001], with a cut-off point of 97.5 % sensitivity and 28.13 % specificity. Notably, the discriminant analysis revealed that the optimal combination of TCONS_00026679, uc002ubt.1, ENST00000411904, and ENST00000547644 distinguished childhood B-ALL patients from T-ALL patients. The following discriminant equation was determined from these results: predicted value of probability (PVP) = 0.284 lnENST00000411904–0.204 lnTCONS_00026679 − 0.335 lnuc002ubt.1 + 0.261 lnENST00000547644–0.019. The ROC curve of the predicted probability showed an increased AUC value, up to 0.9686 [95 % CI: 0.9369–1.000, *P* < 0.001] with 90.7 % sensitivity and 92.19 % specificity at the cut-off point of -0.5700. We have also provided the maximum Likelihood ratio (Table S2). Together, these differential lncRNAs may serve as potential biomarkers in childhood B-ALL and T-ALL patients.

### Specific lncRNA expression is associated with total 8-year leukemia-free survival in childhood ALL

**Fig. 3:**
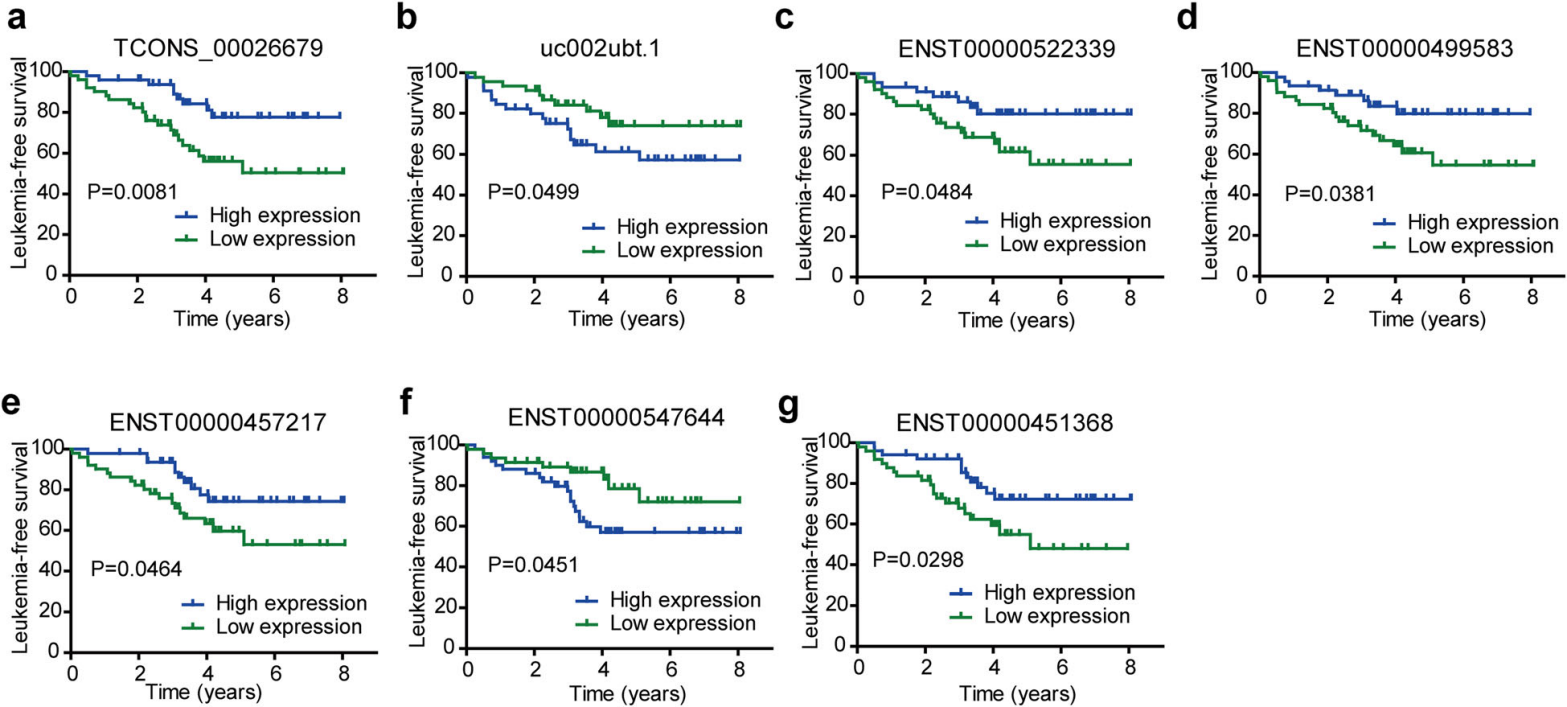
Survival in childhood ALL with specific lncRNA expression. The samples were clustered into high expression and low expression groups according to the median expression of each lncRNA. The 8-year leukemia-free survival of patients with high expression of TCONS_00026679 (**A**), high uc002ubt.1 (**B**), ENST00000522339 (**C**), ENST00000499583 (**D**), ENST00000457217 (**E**), ENST00000547644 (**F**) and ENST00000451368 (**G**)

To further investigate whether the levels of specific lncRNAs can be used to monitor the post-therapeutic progression, all ten validated aberrant lncRNAs were used to perform a Kaplan-Meier analysis with a log-rank test. Here, we compared the expression patterns of these lncRNAs to the clinical outcomes of childhood ALL in an 8-year follow-up survey. The full set of 102 samples was clustered into high expression and low expression groups with respect to the median expression of each lncRNA. As shown in Fig. 3, the 8-year leukemia-free survival of patients with high TCONS_00026679 (*p* = 0.0081), ENST00000522339 (*p* = 0.0484), ENST00000499583 (*p* = 0.0381), ENST00000457217 (*p* = 0.0464), and ENST00000451368 (*p* = 0.0298) expression levels was significantly higher than that in patients with low expression levels of these lncRNAs, while the 8-year leukemia-free survival of patients with high uc002ubt.1 (*p* = 0.0499) and ENST00000547644 (*p* = 0.0451) expression levels was markedly lower than that in the low expression group. Notably, high levels of these lncRNAs at diagnosis were correlated with distinct 8-year leukemia-free survival rates, suggesting that different lncRNAs may have different regulatory functions that convey variable survival in childhood ALL. Taken together, these results indicate that the differential expression of the lncRNAs was significantly associated with poor outcomes in childhood ALL patients, indicating that these lncRNAs represent potential biomarkers for the prognosis of childhood ALL.

### Regulation network of differentially expressed lncRNAs enriched in the pathways and processes of B-ALL and T-ALL

The aforementioned results showed that highly characteristic lncRNAs can be used to monitor the post-therapeutic progression in leukemia. We next endeavored to explore the biological processes of lncRNAs dysregulated in both B-ALL and T-ALL. Therefore, we further performed mRNA microarrays with the same samples used above for the lncRNA array, as shown in Fig. S4A (fold-change > 2.0). The top 65 differentially expressed mRNAs are displayed in Fig. S4B. The most differentially expressed genes were HLA (human leukocyte antigen) genes (HLA-DRA, HLA-DQB1, HLA-DMB, and HLA-DMA) and CD (cluster of differentiation) genes, such as CD9, CD84, CD79B, CD72 and CD3G. To further explore the potential functions of aberrantly expressed lncRNAs, we investigated the expression relationship of dysregulated lncRNAs and genes by spearman correlation analysis. As shown in Fig. S4C, a number of dysregulated lncRNAs and genes have very high expression correlation (Spearman r > 0.5 or <-0.5) and the highest density of Spearman r was near ± 0.5. The closely related expression profiles of aberrant lncRNAs and genes may suggest underlying molecular regulation correlations among them. Subsequently, a Gene Ontology (GO) analysis of the dysregulated lncRNA/mRNA co-expression (Spearman r > 0.9) was used to cluster thousands of transcripts into phenotypically relevant co-expression modules (Table S3). These results revealed that the dysregulated genes belong to several biological process clusters, including proteins involved in apoptosis, immune response, lymphocyte differentiation, cell proliferation, hemopoiesis, and regulation of leukocyte-mediated cytotoxicity, indicating that the dysregulated lncRNAs may play similar roles in these enriched pathways in the progression of B-ALL and T-ALL.

**Fig. 4:**
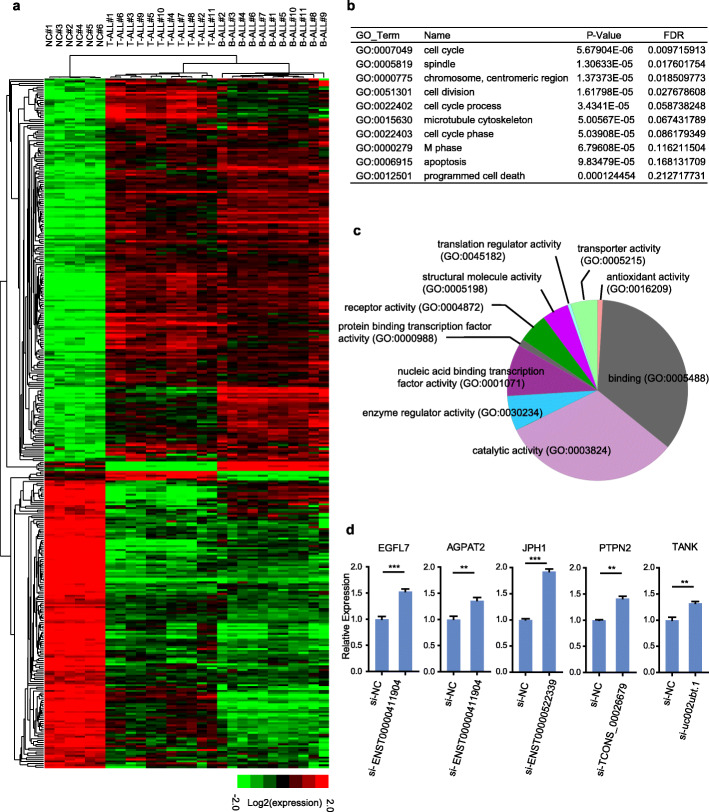
Regulation network of differentially expressed lncRNAs. **A** Genome sites with a set of abnormally adjacent lncRNAs. **B** The top 10 significant GO groups enriched with adjacent genes. **C** The pie chart shows the most significant classes of adjacent genes. **D** The adjacent genes EGFL7, AGPAT2, JPH1, PTPN2, and TANK were significantly upregulated when we knocked down their neighbouring lncRNAs TCONS_00026679, uc002ubt.1, ENST00000522339 and ENST00000411904 in Jurkat cells

Current studies have shown that many lncRNAs target neighborhood coding genes and contribute to similar functional pathways, and gene loci might play important roles in many diseases. We further screened adjacent genes, which are also differentially expressed in the B-ALL samples compared to the T-ALL samples (Fig. 4A), of the dysregulated lncRNAs using stricter conditions (intergenic distance < 10 kb, Table S4) and then analyzed gene functions by GO clustering. The biological process clustering results are listed in Table S5. Figure 4B shows the top 10 significant GO groups of the adjacent genes, for example, cell cycle, cell division and apoptosis. Moreover, molecular function analysis was used to explore the different potential molecular mechanisms underlying the biological process between B-ALL and T-ALL. The pie chart shows the most significant classes of adjacent genes, suggesting that lncRNAs may function on these adjacent genes and contribute to the formation and progression of B-ALL and T-ALL (Fig. 4C).

To further validate the adjacent gene regulation of lncRNAs on adjacent genes, we selected ectopically expressed lncRNAs, which had been previously validated to be abnormally expressed in B-ALL and T-ALL (Fig. 1B-G), to survey their gene loci functions. In this work, we performed an siRNA-mediated knockdown study, and a qRT-PCR assay showed that the interference efficiency of the siRNAs specifically targeting the junction sequence of these lncRNAs was high when they were transfected into both SUP-B15 and Jurkat cells (Fig. S5). The adjacent genes, such as EGFL7 [29], AGPAT2 [30], JPH1[31], PTPN2 [32], and TANK [33], which were reported to play important roles in leukemogenesis, were significantly upregulated when we knocked down their neighbouring lncRNAs TCONS_00026679, uc002ubt.1, ENST00000522339, and ENST00000411904 in Jurkat (Fig. 4D). These results demonstrate that lncRNAs might be involved in different biological pathways that contribute to the development of B-ALL and T-ALL. Further studies are necessary to confirm the correlation between lncRNAs and mRNAs in leukemia pathogenesis.

### Dysregulated lncRNAs exert different functions in cell proliferation and dexamethasone-induced apoptosis

**Fig. 5:**
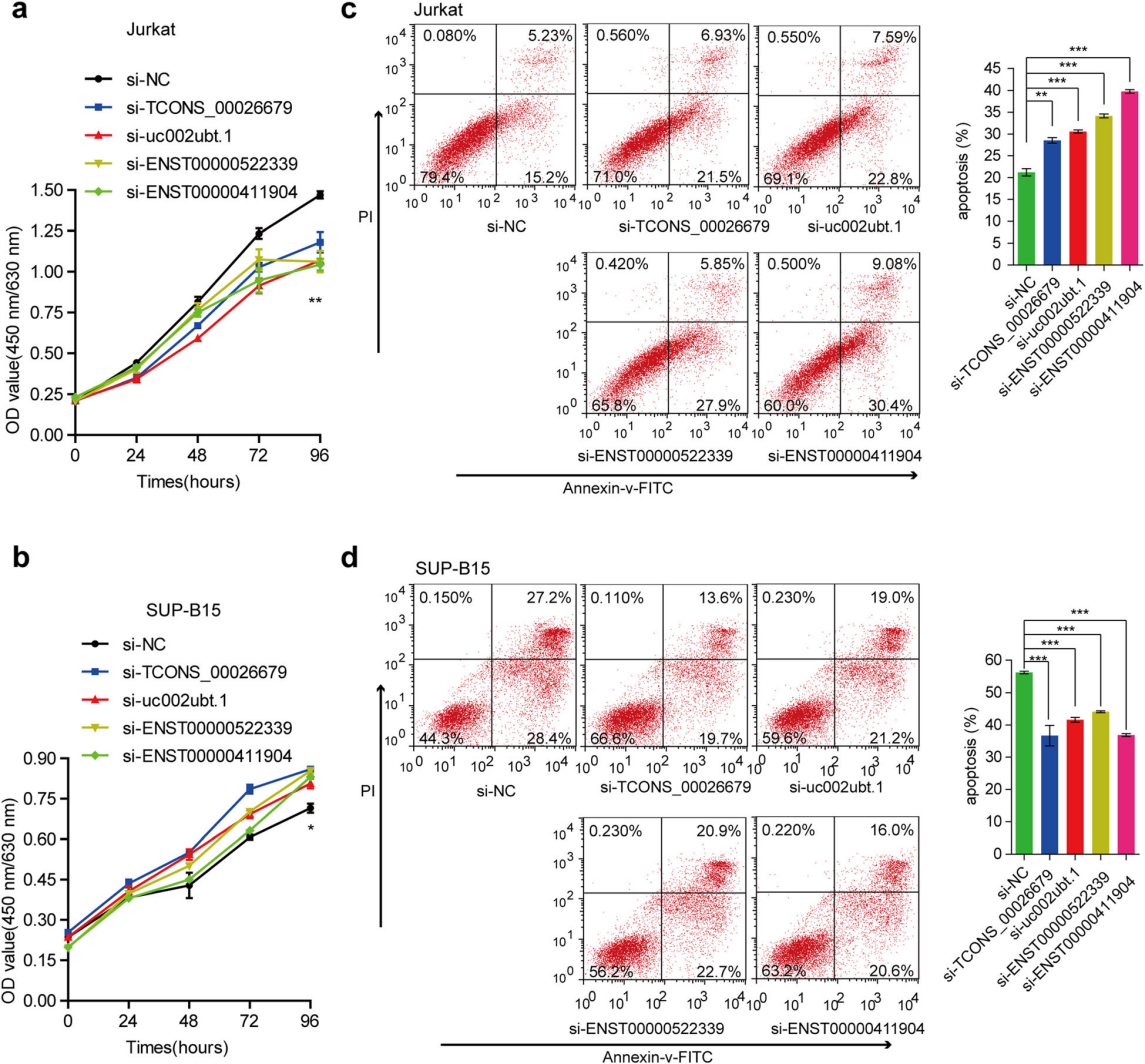
Different biological functions of dysregulated lncRNAs. Using a CCK-8 assay, we found that knocking down these lncRNAs inhibited cell proliferation of Jurkat cells in a time-dependent manner (**A**), whereas they can promote this process in SUP-B15 cells (**B**). In the study of dexamethasone-induced killing and siRNA transfection led to an increase in dexamethasone-triggered apoptosis of Jurkat cells (**C**), and ectopic expression of these four lncRNAs in SUP-B15 cells (**D**) appeared to render a reduction in dexamethasone-triggered apoptosis compared with the control siRNA

The variation in lncRNA expression levels in B-ALL and T-ALL suggested an association between lncRNAs and key functions related to the leukemogenesis of different subsets of ALLs. To explore the different biological functions of the lncRNAs, we selected four ectopically expressed lncRNAs, TCONS_00026679, uc002ubt.1, ENST00000522339, and ENST00000411904, which were suggested to contribute to the development of B-ALL and T-ALL adjacent-regulated gene loci, to investigate their influences on leukemic cell proliferation and apoptosis, which are the pathways with higher scores in the aforementioned GO analysis (Fig. 4B, Tables S3 & S5). Two cell lines, SUP-B15 and Jurkat cells, representing B-ALL and T-ALL, respectively, were used for the in vitro functional analysis. By performing a CCK-8 assay, we found that knockdown of these lncRNAs inhibited cell proliferation of the Jurkat cells in a time-dependent manner (Fig. 5A), whereas they promoted this process in the SUP-B15 cells (Fig. 5B). Moreover, we found that knockdown of these four candidate lncRNAs can slightly affect the cell apoptosis (about 3-5 %) in Jurkat or SUP-B15 cells (Fig. S6). Notably, in the study of dexamethasone-induced killing, siRNA transfection led to an increase in dexamethasone-triggered apoptosis of the Jurkat cells. In particular, the apoptosis rate upon the knockdown of ENST00000411904 reached 40 %, which was significantly higher than that of the negative control (20 %) (Fig. 5C). In contrast, ectopic expression of these four lncRNAs in SUP-B15 cells appeared to render a reduction in dexamethasone-triggered apoptosis compared to a control siRNA (Fig. 5D). This result may indicate that these lncRNAs have different or even opposite functions affecting cell proliferation and apoptosis in B-ALL and T-ALL. Additionally, we performed subcellular fractionation to detect the nuclear/cytoplasm distribution of these four candidate lncRNAs and found these four lncRNAs have different locations in Jurkat and SUP-B15 cells (Fig. S7), which may suggest the different regulation mechanisms of the lncRNAs underlined the opposite functions in B-ALL and T-ALL.

## Discussion

Previous studies have revealed that lncRNAs can function as oncogenes or tumor suppressors to affect cell proliferation, apoptosis, metastasis, etc. Recently, using whole-transcriptome sequencing or microarray methods, some lncRNAs were investigated in childhood B-ALL or T-ALL [25, 34–37]. However, the different lncRNA expression profiles and detailed mechanisms between childhood B-ALL and T-ALL have still not been elucidated. In the present study, we explored the lncRNA expression profile in primary childhood patient samples of B-ALL and T-ALL by microarray. Our results showed that childhood B-ALL and T-ALL have unique expression patterns, and some dysregulated lncRNAs may serve as potential biomarkers for distinguishing ALL subtypes, early diagnosis and prognosis.

Accurate immunotyping of ALL can determine the origin and differentiation stage of leukemia cells, which is of great significance for the diagnosis, treatment and prognosis of leukemia [27, 38, 39]. Clinically, there are so many immunotyping markers for distinguishing ALL in every newly diagnosed patient, as follows: TdT+, CD19+, CD79a+, CD10±, CD 20±, cyIgM±, surface immunoglobulin (sIg)- for B-ALL; and TdT+, cyCD3+, CD7 + T- for ALL [26, 27]. This method is relied heavily on the development of high precision flow cytometry [40]. So, to explore multidimensional detection methods for accurate classification of ALL is meaningful. In the present study, based on genome-wide screening and qRT-PCR validation, we identified that dysregulated lncRNAs are benefit for distinguishing the subtype of ALL. Interestingly, eight lncRNAs have a significant ability to distinguish childhood B-ALL from T-ALL patients with better sensitivity and specificity. Notably, discriminant analysis revealed that the optimal combination of TCONS_00026679, uc002ubt.1, ENST00000411904, and ENST00000547644 has the best potential as a novel biomarker to distinguish childhood B-ALL from T-ALL, with the highest AUC value of 0.9686. We also explored the association of the expression of selected lncRNAs in 102 primary childhood ALL patients and the prognosis of the same patients with an 8-year follow-up. Notably, the Kaplan-Meier analysis showed that expression levels of seven lncRNAs at diagnosis were significantly associated with the outcomes of childhood ALL patients, revealing that these lncRNAs may represent new biomarkers for the prognosis of childhood ALL. However, it should be pointed out that further investigation is necessary to validate the diagnostic or prognostic value of these lncRNAs in a large cohort of samples.

Our mRNA profile showed that the most differentially expressed genes between childhood B-ALL and T-ALL included HLA and CD genes. HLA genes are involved in differentiating self- and non-self- cells in tumors and are candidate genetic susceptibility loci for childhood ALL. It has been reported that the HLA-DRA and HLA-DQB1 genes are associated with the progression of childhood ALL [41, 42], and elevated HLA-DM expression contributes to childhood ALL [43]. CD molecules are commonly used as cell markers to define cells with certain functions or properties and to classify hematological malignancies. A recent ALL study showed that overexpression of CD9 promoted cell migration in B-ALL and was associated with an unfavorable prognosis in ALL patients [44, 45]. CD79B and CD72 were highly expressed in most newly diagnosed B-ALL cases [46, 47]. Further GO analysis revealed that the dysregulated genes contributed to the progression of B-ALL and T-ALL by regulating different biological processes. The regulation of lncRNAs on adjacent genes has been proven to be an important regulatory mechanism of lncRNAs. Our present study highlights the adjacent regulation as one of the crucial mechanisms in ALL. For example, the tyrosine phosphatase PTPN2, an adjacent coding gene of lncRNA TCONS_00026679, has been demonstrated to be a tumor suppressor in T-ALL [32]. We found that lncRNA TCONS_00026679 is highly expressed in T-ALL compared to B-ALL, suggesting that TCONS_00026679 may play an oncogenic role in T-ALL. Notably, knocking down TCONS_00026679 validated that TCONS_00026679 inhibited cell proliferation and promoted apoptosis in T-ALL, confirming its oncogenic roles in T-ALL. Interestingly, the expression level of PTPN2 was significantly increased when TCONS_00026679 was knocked down, supporting the possibility that TCONS_00026679 might function as an oncogene and contribute to the progression of T-ALL via the suppression of the neighbouring tumor suppressor PTPN2. In the study, based on co-expression analysis, adjacent regulation prediction and GO pathway analysis, we investigated the potential regulatory functions of global dysregulated lncRNAs in B-ALL and T-ALL, which provided a regulatory pool for guiding the underlying mechanisms of lncRNAs in childhood ALL progression. However, the majority of lncRNAs involved in childhood ALL have still not been investigated, and their detailed regulatory mechanisms need to be elucidated in the future.

## Conclusions

In summary, this study demonstrated different lncRNA profiles between childhood B-ALL and T-ALL, revealing potential novel biomarkers for the diagnosis and prognosis of childhood B-ALL and T-ALL. In addition, we explored the underlying mechanisms in leukemogenesis. Taken together, these findings will be helpful for better understanding the pathogenesis and providing new diagnosis and treatment strategies for childhood B-ALL and T-ALL.

## Supplementary information

**Additional file 1.**

## Data Availability

Data and materials will be shared.

## Abbreviations

LncRNAs: Long non-coding RNAs
mRNAs: messenger RNAs
ALL: Acute lymphoblastic leukemia
B-ALL: B-cell acute lymphoblastic leukemia
T-ALL: T-cell acute lymphoblastic leukemia
HSCT: Hematopoietic stem cell transplantation
nt: nucleotides
ATCC: American Type Culture Collection
si-RNA: small interfer RNA
CCK-8: Cell Counting Kit-8
CR: Complete remission
GO: Gene Ontology
HLA: Human leukocyte antigens
PVP: Predicted value of probability
ROC: Receiver operating characteristic
