# Supplementary material for: LncRNAs serve as novel biomarkers for diagnosis and prognosis of childhood ALL
Source: Biomark Res. 2021 Jun 10;9:45. doi: 10.1186/s40364-021-00303-x (PMC8193891; doi:10.1186/s40364-021-00303-x)
Supplement: Supplementary file 1 — Additional file 1. [file 40364_2021_303_MOESM1_ESM.docx]

**Supplemental materials**


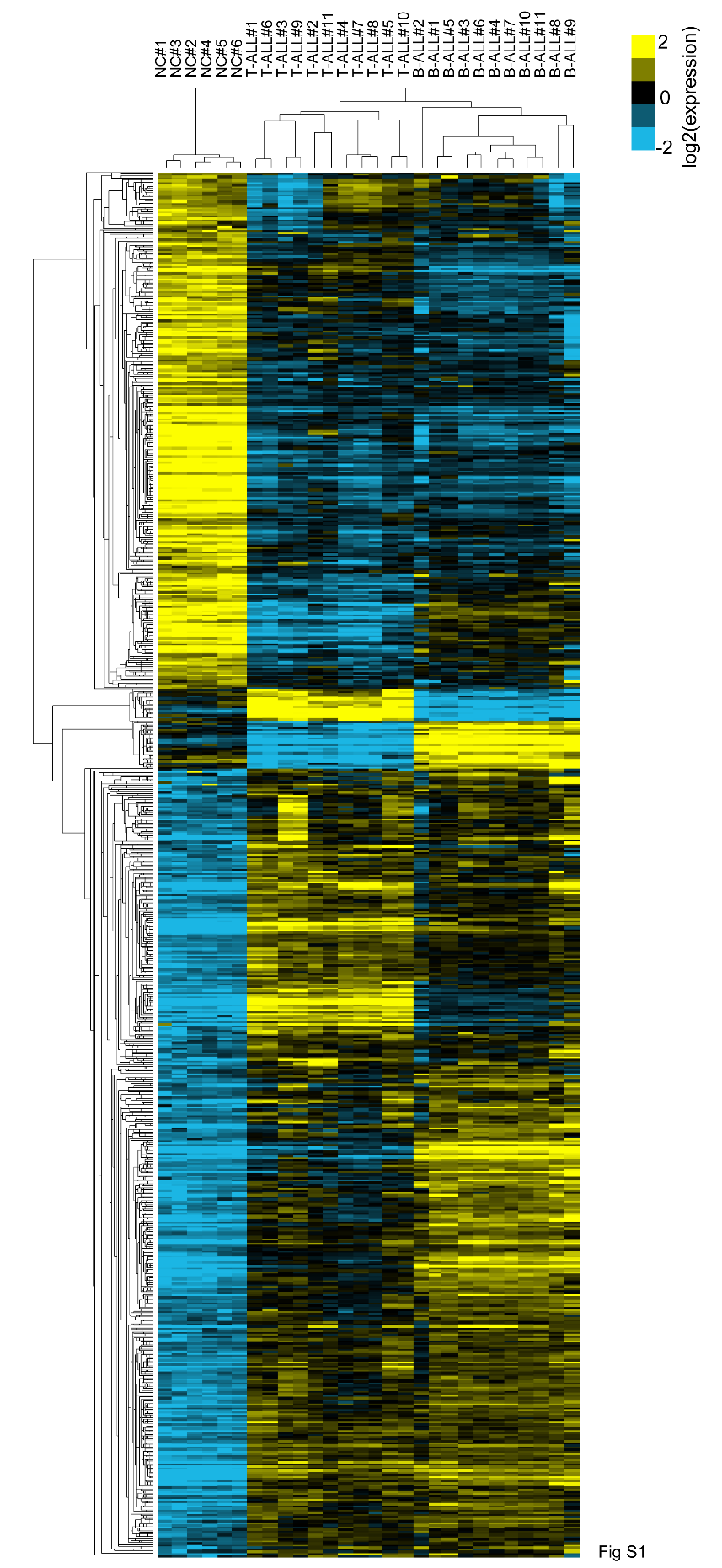


**Fig. S1. Cluster analysis of global aberrantly lncRNA expression in cancerous and adjacent tissues of CCA patients.**


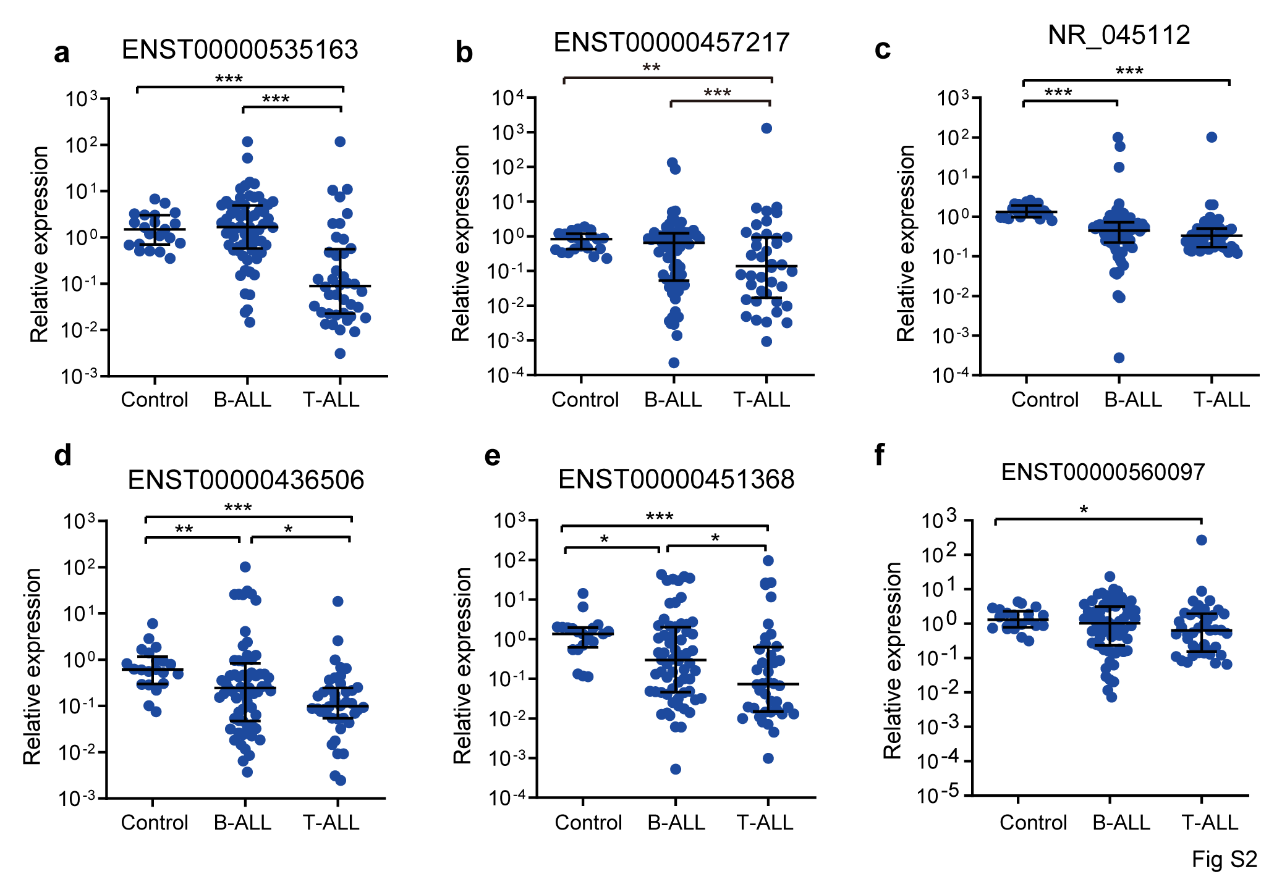


**Fig. S2. The expression of lncRNAs dysregulated in childhood ALL patients.**

The qRT-PCR validation of differentially expressed lncRNA transcripts in childhood ALL patients (64 for B-ALL, 43 for T-ALL and 21 negative control samples). *P < 0.05. **P < 0.01; ***P < 0.001.


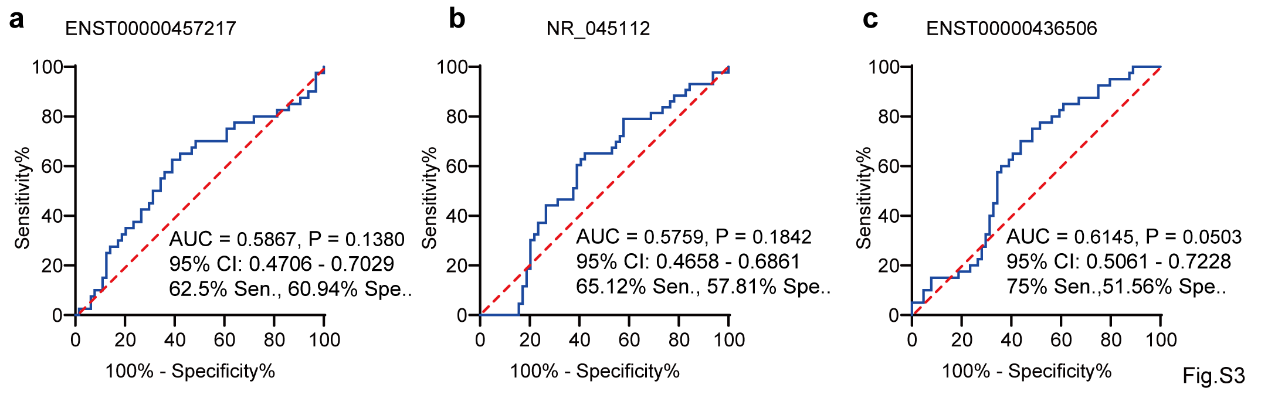


**Fig. S3. Diagnostic value of lncRNAs in childhood B-ALL and T-ALL patients.**

Diagnostic value of lncRNAs for childhood B-ALL and T-ALL patients: ENST00000457217 (**a**), NR_045112 (**b**), and ENST00000436506 (**c**).


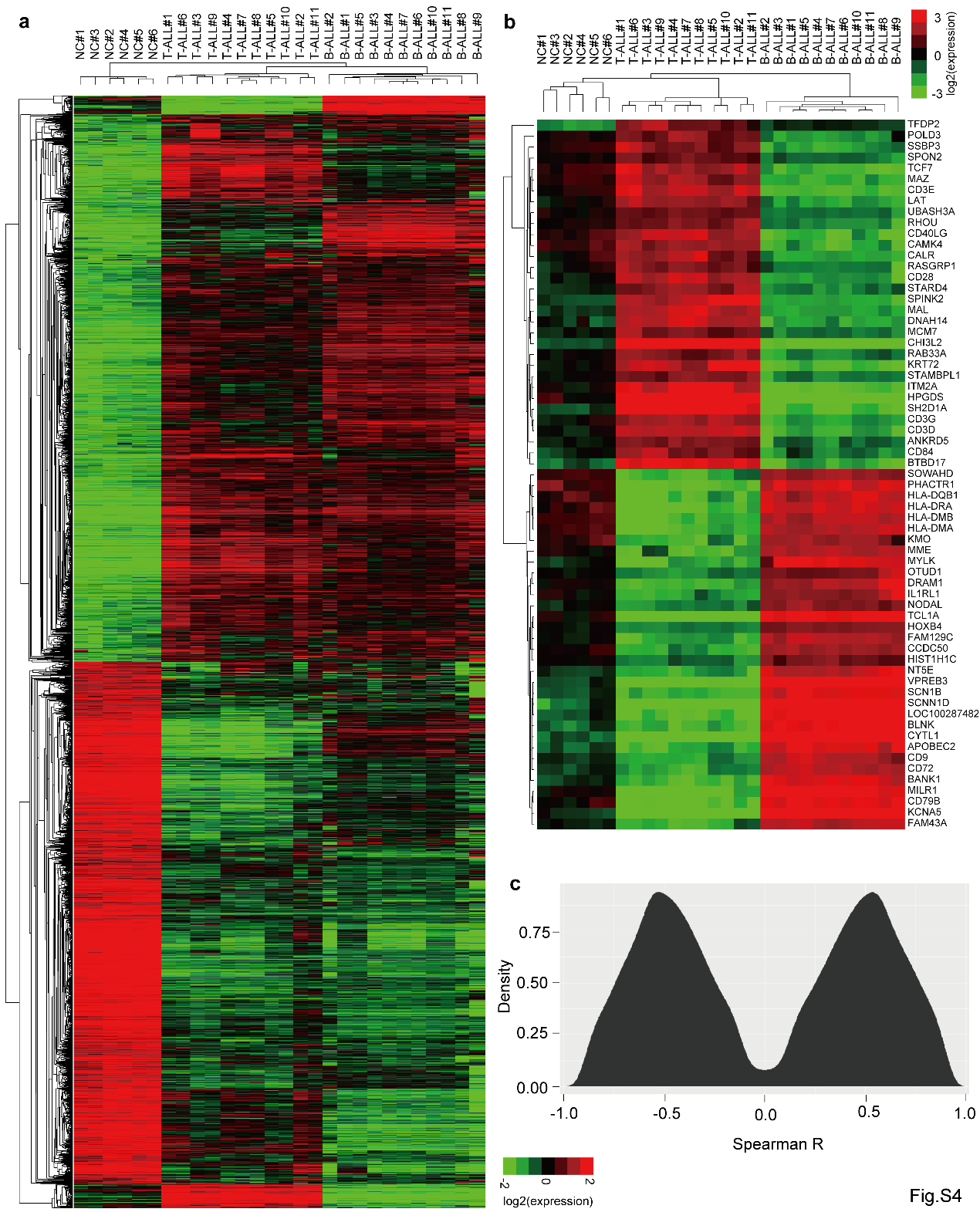


**Fig. S4. The differentially expressed mRNAs in childhood B-ALL and T-ALL.**

**a.** Clustering the global expression profile of aberrant mRNAs among the childhood B-ALL, T-ALL and negative controls (fold-change>2.0). **b.** The top 65 differentially expressed mRNAs are displayed. **c**. The density distribution of spearman rank correlation between the lncRNA/mRNA coexpression.


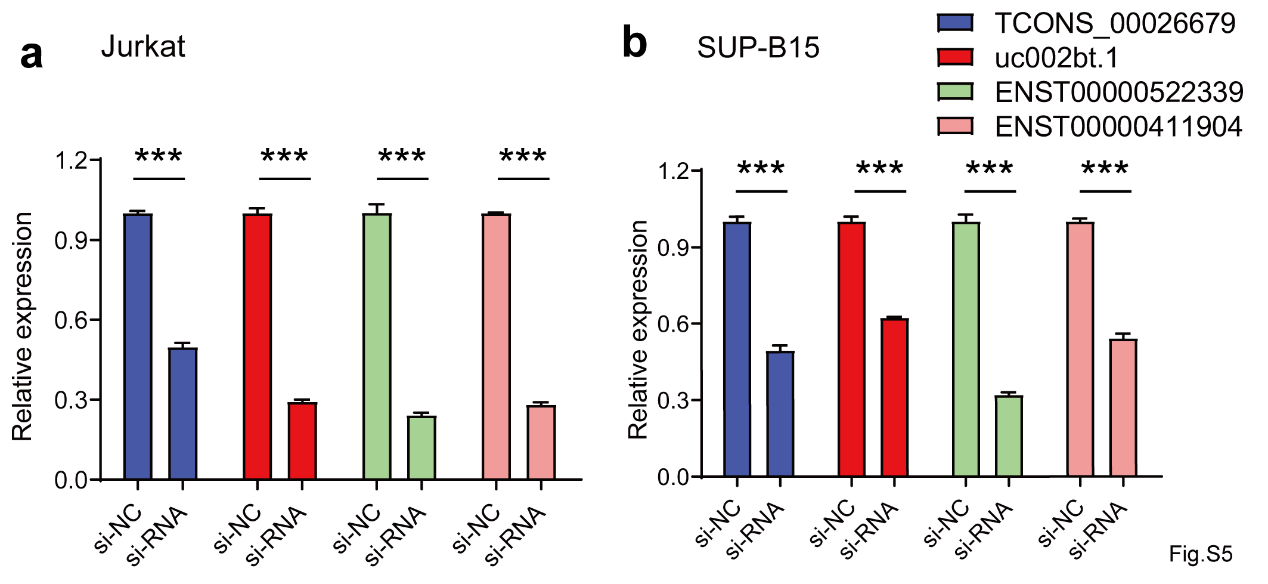


**Fig. S5. A qRT-PCR assay to show the interference efficiency of the siRNAs.**

The specifically targeting these lncRNAs is high, respectively, when transfected them into both Jurkat (**a**) and SUP-B15 (**b**) cell lines. Values are the mean ± SD of three independent experiments. **P < 0.01; ***P < 0.001.


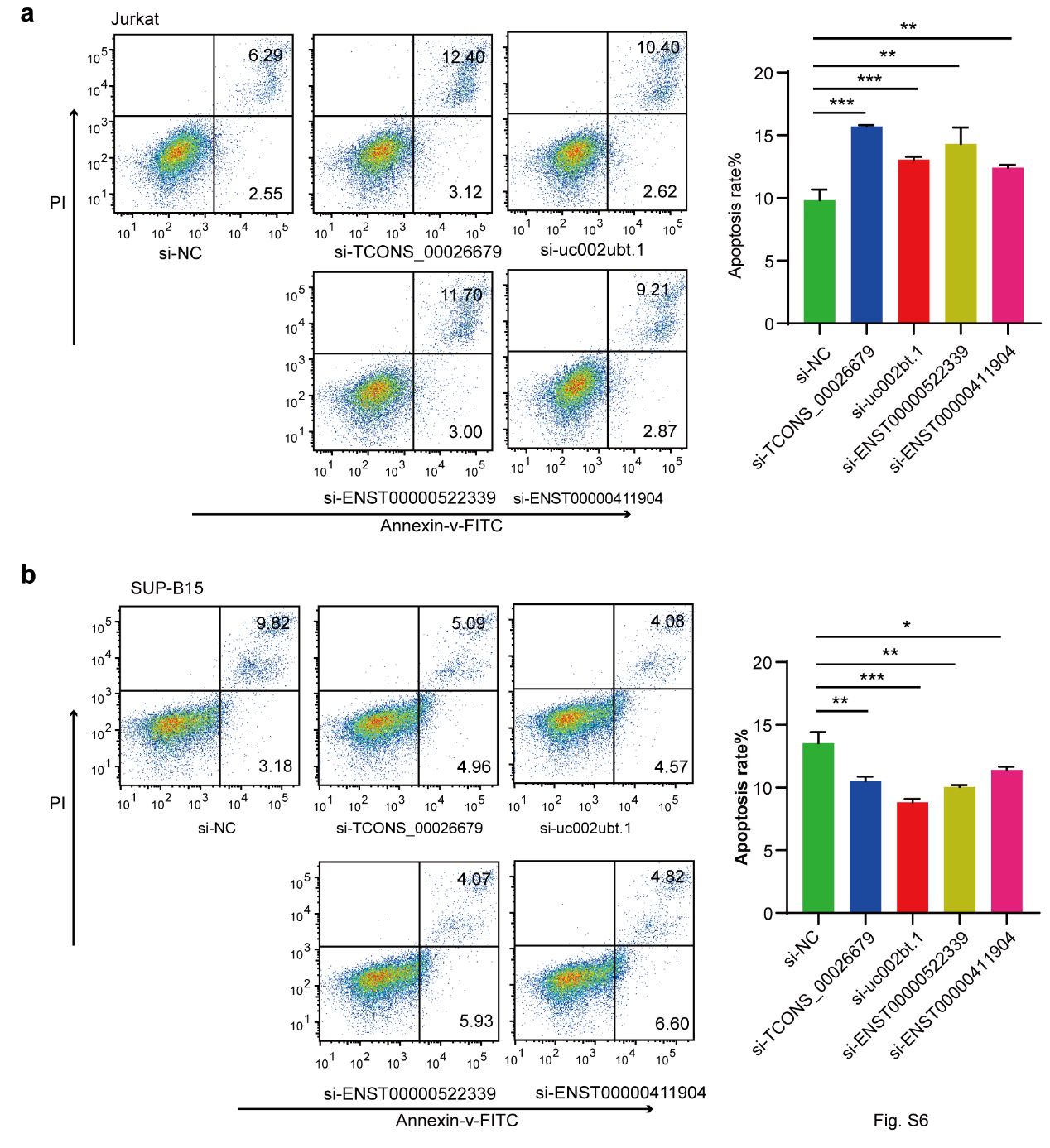


**Fig. S6. Different biological functions of dysregulated lncRNAs.**

Cell apoptosis detection in the lncRNAs silencing Jurkat (**a**) and SUP-B15 cells (**b**). Values are the mean ± SD of three independent experiments. *P < 0.05. **P < 0.01; ***P < 0.001.


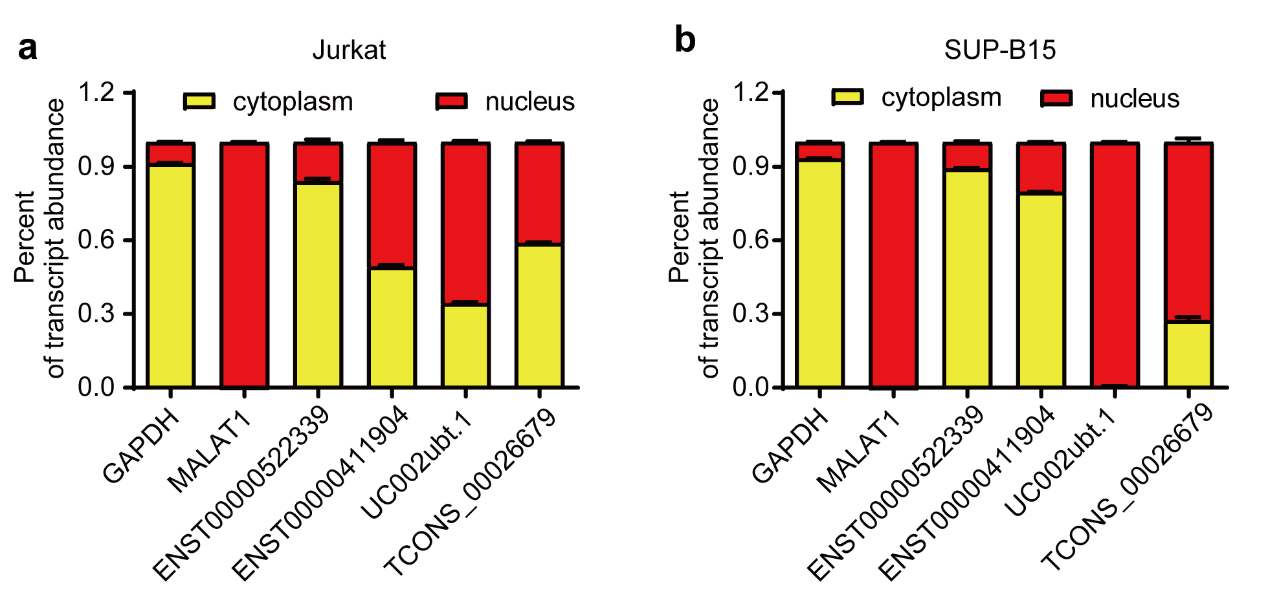


**Fig. S7. Subcellular fractionation of dysregulated lncRNAs.**

qPCR assays showing the relative enrichment of these selected four lncRNAs in cytoplasm and nucleus in Jarkat(**a**) and SUP-B15(**b**) cells. GAPDH acted as cytoplasm control, and MALAT1 nucleus.

**Table S1. Primer and siRNAs sequences are used in this study.**

| **LncRNA** | **Forward** | **Reverse** |
| --- | --- | --- |
| **qPCR primer** |  |  |
| ENST00000411904 | CACTTACTGTTCCCTTGCTC | GGAAACGCTCTGACATCTT |
| NR_045112 | GGCTTCACCATACAACGC | CTGCCAGACTGCCACAGA |
| ENST00000547644 | CAGCTAGTGAGTGGCAGACC | GTGAGGTGGCTGACAGCATA |
| uc002ubt.1 | AGAGCTTCAGCATCCTCGTG | CTGCTGCGTGGAGGATACAA |
| ENST00000436506 | AGAGCTTCAGCATCCTCGTG | CTGCTGCGTGGAGGATACAA |
| ENST00000451368 | ACTGGAAAAGAAGAGAGGGG | AGTCTTCACACACACCTCCAC |
| ENST00000522339 | GAGAGCAGCGACGTGGTAT | GGCTGGCTTCTCCTCCTTA |
| ENST00000457217 | CCCAAATCTTAGTGGCTTACAA | CATTGGTTCTTCCTCCATCAG |
| ENST00000560097 | GGTGGGAGTGACCCGATTT | GCAGCCTCTTGAGCTTGTG |
| TCONS_00026679 | GGAATGCAGGAAGATGGACA | GGGAATGAGTGTTCGTGGG |
| ENST00000535163 | GAGCTGACGCTAAAGACTGAG | CAAGGCTCCTACTGACCATAT |
| ENST00000499583 | ATATGGCTGGCAACACTAGTA | TTTCAAACAGGGAAGGTAGG |
| **siRNA sequences** |  |  |
| TCONS_00026679 | CCUAGGAGAUGAUGUGGAUTT | AUCCACAUCAUCUCCUAGGTT |
| UC002ubt.1 | CCAGCAAGGUUUAUGGUUUTT | AAACCAUAAACCUUGCUGGTT |
| ENST00000522339 | CCUGAUGAGUACAGUCAAATT | UUUGACUGUACUCAUCAGGTT |
| ENST00000411904 | CACUUCCGAAUCCACAGAUTT | AUCUGUGGAUUCGGAAGUGTT |

**Table S2. Maximum Likelihood ratio of the lncRNAs.**

| lncRNA | cut-off point | Sensitivity% | 95% CI | Specificity% | 95% CI | maximum Likelihood ratio |
| --- | --- | --- | --- | --- | --- | --- |
| TCONS_00026679 | > 7.336 | 30 | 18.07% - 45.43% | 93.75 | 85.00% - 97.54% | 4.8 |
| uc002ubt.1 | > 3.982 | 50 | 35.20% - 64.80% | 98.39 | 91.41% - 99.92% | 31 |
| ENST00000522339 | < 0.009324 | 42.5 | 28.51% - 57.80% | 95.31 | 87.10% - 98.72% | 9.067 |
| ENST00000411904 | < 0.1103 | 55 | 39.83% - 69.29% | 96.88 | 89.30% - 99.44% | 17.6 |
| ENST00000547644 | < 0.4075 | 85 | 70.93% - 92.94% | 92.19 | 82.98% - 96.62% | 10.88 |
| ENST00000499583 | < 0.009072 | 35 | 22.13% - 50.49% | 95.08 | 86.51% - 98.66% | 7.117 |
| ENST00000535163 | < 0.02358 | 27.5 | 16.11% - 42.83% | 98.46 | 91.79% - 99.92% | 17.88 |
| ENST00000457217 | < 0.01543 | 25 | 14.19% - 40.19% | 87.5 | 77.23% - 93.53% | 2 |
| NR_045112 | < 0.4022 | 65.12 | 50.17% - 77.58% | 57.81 | 45.61% - 69.13% | 1.543 |
| ENST00000436506 | < 0.005126 | 5 | 0.8884% - 16.50% | 98.44 | 91.67% - 99.92% | 3.2 |
| ENST00000451368 | < 0.01134 | 15 | 7.061% - 29.07% | 95.31 | 87.10% - 98.72% | 3.2 |
| Combination of TCONS_00026679, uc002ubt.1, ENST00000411904, and ENST00000547644 | < -1.080 | 76.74 | 62.26% to 86.85% | 98.44 | 75.38% - 92.54% | 49.12 |

**Table S3. GO clusters of dysregulated genes of dysregulated lncRNAs (spearman r>0.9).**

| **GO_Term** | **name** | **P-Value** | **FDR** |
| --- | --- | --- | --- |
| **GO:0006955** | immune response | 1.02E-19 | 1.73E-16 |
| **GO:0006952** | defense response | 1.09E-07 | 1.85E-04 |
| **GO:0045321** | leukocyte activation | 2.58E-07 | 4.37E-04 |
| **GO:0051249** | regulation of lymphocyte activation | 3.21E-07 | 5.43E-04 |
| **GO:0001775** | cell activation | 7.19E-07 | 1.21E-03 |
| **GO:0051251** | positive regulation of lymphocyte activation | 1.00E-06 | 1.69E-03 |
| **GO:0002694** | regulation of leukocyte activation | 1.30E-06 | 2.20E-03 |
| **GO:0030098** | lymphocyte differentiation | 1.83E-06 | 3.09E-03 |
| **GO:0050865** | regulation of cell activation | 2.43E-06 | 4.11E-03 |
| **GO:0002696** | positive regulation of leukocyte activation | 2.43E-06 | 4.12E-03 |
| **GO:0050867** | positive regulation of cell activation | 3.84E-06 | 6.49E-03 |
| **GO:0042102** | positive regulation of T cell proliferation | 4.42E-06 | 7.48E-03 |
| **GO:0002684** | positive regulation of immune system process | 4.56E-06 | 7.71E-03 |
| **GO:0050671** | positive regulation of lymphocyte proliferation | 4.72E-06 | 7.98E-03 |
| **GO:0070665** | positive regulation of leukocyte proliferation | 5.42E-06 | 9.17E-03 |
| **GO:0032946** | positive regulation of mononuclear cell proliferation | 5.42E-06 | 9.17E-03 |
| **GO:0050863** | regulation of T cell activation | 6.42E-06 | 1.08E-02 |
| **GO:0050870** | positive regulation of T cell activation | 6.86E-06 | 1.16E-02 |
| **GO:0002697** | regulation of immune effector process | 1.07E-05 | 1.81E-02 |
| **GO:0046649** | lymphocyte activation | 1.08E-05 | 1.83E-02 |
| **GO:0050670** | regulation of lymphocyte proliferation | 1.42E-05 | 2.40E-02 |
| **GO:0070663** | regulation of leukocyte proliferation | 1.57E-05 | 2.65E-02 |
| **GO:0032944** | regulation of mononuclear cell proliferation | 1.57E-05 | 2.65E-02 |
| **GO:0030217** | T cell differentiation | 1.68E-05 | 2.84E-02 |
| **GO:0001817** | regulation of cytokine production | 1.80E-05 | 3.05E-02 |
| **GO:0002521** | leukocyte differentiation | 1.89E-05 | 3.20E-02 |
| **GO:0030097** | hemopoiesis | 7.19E-05 | 1.22E-01 |
| **GO:0042110** | T cell activation | 7.35E-05 | 1.24E-01 |
| **GO:0033077** | T cell differentiation in the thymus | 9.32E-05 | 1.57E-01 |
| **GO:0042129** | regulation of T cell proliferation | 1.00E-04 | 1.70E-01 |
| **GO:0002699** | positive regulation of immune effector process | 1.10E-04 | 1.85E-01 |
| **GO:0048534** | hemopoietic or lymphoid organ development | 2.00E-04 | 3.38E-01 |
| **GO:0050778** | positive regulation of immune response | 2.35E-04 | 3.97E-01 |
| **GO:0002504** | antigen processing and presentation of peptide or polysaccharide antigen via MHC class II | 2.52E-04 | 4.24E-01 |
| **GO:0048584** | positive regulation of response to stimulus | 2.70E-04 | 4.56E-01 |
| **GO:0045076** | regulation of interleukin-2 biosynthetic process | 2.89E-04 | 4.88E-01 |
| **GO:0045058** | T cell selection | 2.89E-04 | 4.88E-01 |
| **GO:0002705** | positive regulation of leukocyte mediated immunity | 2.91E-04 | 4.90E-01 |
| **GO:0002708** | positive regulation of lymphocyte mediated immunity | 2.91E-04 | 4.90E-01 |
| **GO:0042035** | regulation of cytokine biosynthetic process | 3.08E-04 | 5.19E-01 |
| **GO:0002706** | regulation of lymphocyte mediated immunity | 3.47E-04 | 5.85E-01 |
| **GO:0002520** | immune system development | 3.68E-04 | 6.21E-01 |
| **GO:0046641** | positive regulation of alpha-beta T cell proliferation | 4.13E-04 | 6.95E-01 |
| **GO:0001912** | positive regulation of leukocyte mediated cytotoxicity | 4.35E-04 | 7.32E-01 |
| **GO:0042552** | myelination | 4.95E-04 | 8.34E-01 |
| **GO:0019882** | antigen processing and presentation | 6.21E-04 | 1.05E+00 |
| **GO:0002703** | regulation of leukocyte mediated immunity | 6.74E-04 | 1.13E+00 |
| **GO:0031343** | positive regulation of cell killing | 7.41E-04 | 1.24E+00 |
| **GO:0007272** | ensheathment of neurons | 7.93E-04 | 1.33E+00 |
| **GO:0008366** | axon ensheathment | 7.93E-04 | 1.33E+00 |
| **GO:0001910** | regulation of leukocyte mediated cytotoxicity | 8.70E-04 | 1.46E+00 |
| **GO:0046640** | regulation of alpha-beta T cell proliferation | 1.33E-03 | 2.23E+00 |
| **GO:0045061** | thymic T cell selection | 1.33E-03 | 2.23E+00 |
| **GO:0045086** | positive regulation of interleukin-2 biosynthetic process | 1.33E-03 | 2.23E+00 |
| **GO:0007159** | leukocyte adhesion | 1.35E-03 | 2.26E+00 |
| **GO:0031341** | regulation of cell killing | 1.35E-03 | 2.26E+00 |
| **GO:0042108** | positive regulation of cytokine biosynthetic process | 1.47E-03 | 2.45E+00 |
| **GO:0031349** | positive regulation of defense response | 1.74E-03 | 2.89E+00 |
| **GO:0032663** | regulation of interleukin-2 production | 1.76E-03 | 2.93E+00 |
| **GO:0006928** | cell motion | 1.78E-03 | 2.96E+00 |
| **GO:0050864** | regulation of B cell activation | 1.93E-03 | 3.21E+00 |
| **GO:0045088** | regulation of innate immune response | 2.49E-03 | 4.12E+00 |
| **GO:0019228** | regulation of action potential in neuron | 2.49E-03 | 4.12E+00 |
| **GO:0045954** | positive regulation of natural killer cell mediated cytotoxicity | 2.51E-03 | 4.16E+00 |
| **GO:0002717** | positive regulation of natural killer cell mediated immunity | 2.51E-03 | 4.16E+00 |
| **GO:0001959** | regulation of cytokine-mediated signaling pathway | 2.51E-03 | 4.16E+00 |
| **GO:0050871** | positive regulation of B cell activation | 2.52E-03 | 4.18E+00 |
| **GO:0022610** | biological adhesion | 2.82E-03 | 4.66E+00 |
| **GO:0007155** | cell adhesion | 2.84E-03 | 4.69E+00 |
| **GO:0002715** | regulation of natural killer cell mediated immunity | 3.01E-03 | 4.97E+00 |
| **GO:0042269** | regulation of natural killer cell mediated cytotoxicity | 3.01E-03 | 4.97E+00 |
| **GO:0050850** | positive regulation of calcium-mediated signaling | 3.01E-03 | 4.97E+00 |
| **GO:0006954** | inflammatory response | 4.83E-03 | 7.85E+00 |
| **GO:0050848** | regulation of calcium-mediated signaling | 4.86E-03 | 7.90E+00 |
| **GO:0009611** | response to wounding | 5.58E-03 | 9.02E+00 |
| **GO:0002700** | regulation of production of molecular mediator of immune response | 5.60E-03 | 9.05E+00 |
| **GO:0045059** | positive thymic T cell selection | 6.06E-03 | 9.76E+00 |
| **GO:0008284** | positive regulation of cell proliferation | 6.31E-03 | 1.02E+01 |
| **GO:0001508** | regulation of action potential | 6.73E-03 | 1.08E+01 |
| **GO:0042981** | regulation of apoptosis | 7.48E-03 | 1.19E+01 |
| **GO:0043067** | regulation of programmed cell death | 8.40E-03 | 1.33E+01 |
| **GO:0045089** | positive regulation of innate immune response | 8.43E-03 | 1.33E+01 |
| **GO:0010941** | regulation of cell death | 8.77E-03 | 1.38E+01 |
| **GO:0043368** | positive T cell selection | 1.01E-02 | 1.58E+01 |
| **GO:0046635** | positive regulation of alpha-beta T cell activation | 1.03E-02 | 1.60E+01 |
| **GO:0002637** | regulation of immunoglobulin production | 1.03E-02 | 1.60E+01 |
| **GO:0007265** | Ras protein signal transduction | 1.04E-02 | 1.61E+01 |
| **GO:0030155** | regulation of cell adhesion | 1.04E-02 | 1.62E+01 |
| **GO:0042113** | B cell activation | 1.07E-02 | 1.66E+01 |
| **GO:0019220** | regulation of phosphate metabolic process | 1.15E-02 | 1.78E+01 |
| **GO:0051174** | regulation of phosphorus metabolic process | 1.15E-02 | 1.78E+01 |
| **GO:0030888** | regulation of B cell proliferation | 1.26E-02 | 1.93E+01 |
| **GO:0002683** | negative regulation of immune system process | 1.52E-02 | 2.28E+01 |
| **GO:0002824** | positive regulation of adaptive immune response based on somatic recombination of immune receptors built from immunoglobulin superfamily domains | 1.52E-02 | 2.28E+01 |
| **GO:0002822** | regulation of adaptive immune response based on somatic recombination of immune receptors built from immunoglobulin superfamily domains | 1.56E-02 | 2.33E+01 |
| **GO:0009968** | negative regulation of signal transduction | 1.60E-02 | 2.38E+01 |
| **GO:0002819** | regulation of adaptive immune response | 1.66E-02 | 2.46E+01 |
| **GO:0002821** | positive regulation of adaptive immune response | 1.66E-02 | 2.47E+01 |
| **GO:0042325** | regulation of phosphorylation | 1.74E-02 | 2.57E+01 |
| **GO:0050900** | leukocyte migration | 1.76E-02 | 2.59E+01 |
| **GO:0050854** | regulation of antigen receptor-mediated signaling pathway | 1.80E-02 | 2.64E+01 |
| **GO:0007242** | intracellular signaling cascade | 1.99E-02 | 2.88E+01 |
| **GO:0006968** | cellular defense response | 2.20E-02 | 3.14E+01 |
| **GO:0016477** | cell migration | 2.38E-02 | 3.34E+01 |
| **GO:0007249** | I-kappaB kinase/NF-kappaB cascade | 2.45E-02 | 3.42E+01 |
| **GO:0046634** | regulation of alpha-beta T cell activation | 2.48E-02 | 3.46E+01 |
| **GO:0032103** | positive regulation of response to external stimulus | 2.57E-02 | 3.56E+01 |
| **GO:0042063** | gliogenesis | 2.71E-02 | 3.71E+01 |
| **GO:0002711** | positive regulation of T cell mediated immunity | 2.76E-02 | 3.77E+01 |
| **GO:0007243** | protein kinase cascade | 3.04E-02 | 4.06E+01 |
| **GO:0002252** | immune effector process | 3.05E-02 | 4.08E+01 |
| **GO:0042391** | regulation of membrane potential | 3.05E-02 | 4.08E+01 |
| **GO:0010648** | negative regulation of cell communication | 3.09E-02 | 4.11E+01 |
| **GO:0045577** | regulation of B cell differentiation | 3.12E-02 | 4.15E+01 |
| **GO:0050730** | regulation of peptidyl-tyrosine phosphorylation | 3.13E-02 | 4.16E+01 |
| **GO:0043406** | positive regulation of MAP kinase activity | 3.35E-02 | 4.38E+01 |
| **GO:0042127** | regulation of cell proliferation | 3.60E-02 | 4.62E+01 |
| **GO:0043405** | regulation of MAP kinase activity | 3.78E-02 | 4.78E+01 |
| **GO:0045859** | regulation of protein kinase activity | 4.09E-02 | 5.06E+01 |
| **GO:0007599** | hemostasis | 4.13E-02 | 5.10E+01 |
| **GO:0045860** | positive regulation of protein kinase activity | 4.28E-02 | 5.23E+01 |
| **GO:0006693** | prostaglandin metabolic process | 4.30E-02 | 5.24E+01 |
| **GO:0006692** | prostanoid metabolic process | 4.30E-02 | 5.24E+01 |
| **GO:0008283** | cell proliferation | 4.37E-02 | 5.30E+01 |
| **GO:0007169** | transmembrane receptor protein tyrosine kinase signaling pathway | 4.38E-02 | 5.31E+01 |
| **GO:0048870** | cell motility | 4.42E-02 | 5.34E+01 |
| **GO:0051674** | localization of cell | 4.42E-02 | 5.34E+01 |
| **GO:0050727** | regulation of inflammatory response | 4.43E-02 | 5.35E+01 |
| **GO:0019226** | transmission of nerve impulse | 4.45E-02 | 5.36E+01 |
| **GO:0030890** | positive regulation of B cell proliferation | 4.72E-02 | 5.58E+01 |
| **GO:0050731** | positive regulation of peptidyl-tyrosine phosphorylation | 4.90E-02 | 5.72E+01 |
| **GO:0043549** | regulation of kinase activity | 5.00E-02 | 5.80E+01 |

**Table S4.** **Adjacent dysregulated genes of dysregulated lncRNAs.**

| **mRNA** | **Chromosome** | **mRNA_strand** | **lncRNA_symbol** | **lncRNA_start** | **lncRNA_end** | **lncRNA_strand** |
| --- | --- | --- | --- | --- | --- | --- |
| **SRSF10** | chr1 | - | NR_034035 | 24292936 | 24306953 | - |
| **DFFA** | chr1 | - | TCONS_00001938 | 10516602 | 10520093 | + |
| **DFFA** | chr1 | - | ENST00000424487 | 10518611 | 10519395 | + |
| **DFFA** | chr1 | - | NR_037187 | 10490803 | 10512060 | + |
| **G0S2** | chr1 | + | ENST00000445272 | 209834708 | 209848592 | - |
| **CACYBP** | chr1 | + | TCONS_00002518 | 174966628 | 174968522 | - |
| **CACYBP** | chr1 | + | TCONS_00002518 | 174966628 | 174968522 | - |
| **RFX5** | chr1 | - | ENST00000455503 | 151319442 | 151320503 | + |
| **DYRK3** | chr1 | + | ENST00000425161 | 206807993 | 206808967 | - |
| **RABGAP1L** | chr1 | + | ENST00000452442 | 174904083 | 174923398 | - |
| **SNX27** | chr1 | + | uc001eyq.1 | 151670318 | 151670850 | - |
| **ADAMTSL4** | chr1 | + | ENST00000442435 | 150521039 | 150530949 | - |
| **TTF2** | chr1 | + | TCONS_00002142 | 117647168 | 117647504 | + |
| **EFCAB7** | chr1 | + | NR_045147 | 63906440 | 63988944 | - |
| **APITD1** | chr1 | + | NR_036462 | 10490803 | 10502872 | + |
| **APITD1** | chr1 | + | NR_037187 | 10490803 | 10512060 | + |
| **LYST** | chr1 | - | ENST00000446945 | 235989881 | 235991238 | - |
| **ARID4B** | chr1 | - | NR_027762 | 235294497 | 235324571 | - |
| **TOMM20** | chr1 | - | NR_027762 | 235294497 | 235324571 | - |
| **FAIM3** | chr1 | - | TCONS_00002569 | 207096851 | 207097961 | - |
| **FAIM3** | chr1 | - | TCONS_00001785 | 207097099 | 207097924 | - |
| **FAM72D** | chr1 | - | NR_034178 | 143915747 | 144094477 | + |
| **SLC16A1** | chr1 | - | uc001edd.3 | 113499070 | 113511603 | + |
| **SLC16A1** | chr1 | - | ENST00000420168 | 113499141 | 113511020 | + |
| **CTBS** | chr1 | - | ENST00000554297 | 84982042 | 85018808 | + |
| **SRSF10** | chr1 | - | NR_034035 | 24292936 | 24306953 | - |
| **FAIM3** | chr1 | - | TCONS_00002569 | 207096851 | 207097961 | - |
| **FAIM3** | chr1 | - | TCONS_00001785 | 207097099 | 207097924 | - |
| **FAIM3** | chr1 | - | TCONS_00002569 | 207096851 | 207097961 | - |
| **FAIM3** | chr1 | - | TCONS_00001785 | 207097099 | 207097924 | - |
| **APITD1-CORT** | chr1 | + | TCONS_00001938 | 10516602 | 10520093 | + |
| **APITD1-CORT** | chr1 | + | ENST00000424487 | 10518611 | 10519395 | + |
| **APITD1-CORT** | chr1 | + | NR_036462 | 10490803 | 10502872 | + |
| **APITD1-CORT** | chr1 | + | NR_037187 | 10490803 | 10512060 | + |
| **SRSF10** | chr1 | - | NR_034035 | 24292936 | 24306953 | - |
| **RCAN3** | chr1 | + | ENST00000445805 | 24863137 | 24865371 | - |
| **RCAN3** | chr1 | + | uc021ojb.1 | 24822822 | 24828850 | - |
| **HYI** | chr1 | - | ENST00000444386 | 43919597 | 43922666 | + |
| **HYI** | chr1 | - | ENST00000444386 | 43919597 | 43922666 | + |
| **DEPDC1** | chr1 | - | ENST00000428732 | 68962358 | 69004310 | + |
| **CNN3** | chr1 | - | ENST00000452846 | 95393121 | 95428826 | + |
| **FCGR1B** | chr1 | - | NR_045213 | 120926127 | 120935944 | - |
| **FCGR1B** | chr1 | - | NR_045213 | 120926127 | 120935944 | - |
| **CERS2** | chr1 | - | ENST00000561111 | 150937735 | 150938734 | + |
| **MUC1** | chr1 | - | ENST00000453136 | 155168374 | 155175286 | + |
| **CD48** | chr1 | - | ENST00000443928 | 160643135 | 160653612 | + |
| **GORAB** | chr1 | + | NR_027397 | 170501262 | 170522974 | + |
| **RHOU** | chr1 | + | NR_037962 | 228780393 | 228882416 | + |
| **RBM34** | chr1 | - | NR_027762 | 235294497 | 235324571 | - |
| **EFCAB2** | chr1 | + | NR_026587 | 245133630 | 245288530 | + |
| **EFCAB2** | chr1 | + | NR_026587 | 245133630 | 245288530 | + |
| **DARS2** | chr1 | + | ENST00000434796 | 173833117 | 173834039 | - |
| **DARS2** | chr1 | + | NR_037605 | 173832385 | 173833079 | + |
| **TSEN15** | chr1 | + | NR_023349 | 184020810 | 184043344 | + |
| **TNFRSF14** | chr1 | + | TCONS_00001378 | 2499406 | 2500424 | - |
| **TNFRSF14** | chr1 | + | NR_037844 | 2487207 | 2488450 | - |
| **LRP8** | chr1 | - | uc001cvn.1 | 53793904 | 53802889 | + |
| **LRP8** | chr1 | - | ENST00000445039 | 53793920 | 53802890 | + |
| **LRP8** | chr1 | - | NR_038953 | 53704281 | 53708455 | + |
| **LRP8** | chr1 | - | ENST00000450469 | 53757462 | 53757810 | - |
| **GBP5** | chr1 | - | ENST00000437128 | 89726264 | 89735437 | + |
| **JAK1** | chr1 | - | ENST00000447748 | 65437907 | 65458695 | - |
| **SRSF10** | chr1 | - | NR_034035 | 24292936 | 24306953 | - |
| **MTX1** | chr1 | + | ENST00000453136 | 155168374 | 155175286 | + |
| **LRP8** | chr1 | - | uc001cvn.1 | 53793904 | 53802889 | + |
| **LRP8** | chr1 | - | ENST00000445039 | 53793920 | 53802890 | + |
| **LRP8** | chr1 | - | NR_038953 | 53704281 | 53708455 | + |
| **LRP8** | chr1 | - | ENST00000450469 | 53757462 | 53757810 | - |
| **EXOSC10** | chr1 | - | ENST00000435388 | 11159731 | 11162157 | + |
| **EXOSC10** | chr1 | - | ENST00000452378 | 11128527 | 11133154 | + |
| **ARL8A** | chr1 | - | NR_037664 | 202116140 | 202130716 | - |
| **ADAMTSL4** | chr1 | + | ENST00000442435 | 150521039 | 150530949 | - |
| **ITGB3BP** | chr1 | - | NR_045147 | 63906440 | 63988944 | - |
| **TRIM45** | chr1 | - | TCONS_00002142 | 117647168 | 117647504 | + |
| **CHIT1** | chr1 | - | NR_045785 | 203185206 | 203198860 | - |
| **SELL** | chr1 | - | NR_029467 | 169659805 | 169677997 | - |
| **YIPF1** | chr1 | - | NR_036640 | 54317391 | 54355487 | - |
| **DHRS9** | chr2 | + | ENST00000432133 | 169957252 | 169957720 | + |
| **ARHGAP25** | chr2 | + | ENST00000457602 | 69062135 | 69064856 | + |
| **CDC42EP3** | chr2 | - | ENST00000419425 | 37827278 | 37873841 | + |
| **ARHGAP25** | chr2 | + | ENST00000457602 | 69062135 | 69064856 | + |
| **OSGEPL1** | chr2 | - | ENST00000520651 | 190627429 | 190630282 | + |
| **ZNF513** | chr2 | - | ENST00000412749 | 27580054 | 27590489 | + |
| **PRKRA** | chr2 | - | ENST00000450044 | 179278694 | 179296068 | + |
| **PRKRA** | chr2 | - | ENST00000420672 | 179278665 | 179295551 | + |
| **PRKRA** | chr2 | - | ENST00000453026 | 179278720 | 179303866 | + |
| **PRKRA** | chr2 | - | ENST00000436616 | 179278725 | 179298716 | + |
| **PRKRA** | chr2 | - | uc002umh.1 | 179296271 | 179298257 | - |
| **FAM126B** | chr2 | - | ENST00000332935 | 201828021 | 201843705 | + |
| **CERKL** | chr2 | - | NR_027689 | 182401400 | 182521834 | - |
| **CLHC1** | chr2 | - | TCONS_00004256 | 55378212 | 55397546 | - |
| **CLHC1** | chr2 | - | TCONS_00004256 | 55378212 | 55397546 | - |
| **TSN** | chr2 | + | NR_048558 | 122513120 | 122525428 | + |
| **TSN** | chr2 | + | NR_048556 | 122513120 | 122525428 | + |
| **BCL11A** | chr2 | - | uc002sag.3 | 60751623 | 60755350 | - |
| **BCL11A** | chr2 | - | uc002sag.3 | 60751623 | 60755350 | - |
| **DTNB** | chr2 | - | ENST00000431557 | 25592041 | 25598714 | - |
| **DTYMK** | chr2 | - | NR_033255 | 242615156 | 242626383 | - |
| **ARHGAP25** | chr2 | + | ENST00000457602 | 69062135 | 69064856 | + |
| **SPOPL** | chr2 | + | ENST00000458007 | 139326659 | 139331818 | + |
| **SPOPL** | chr2 | + | ENST00000449869 | 139326661 | 139328644 | + |
| **MXD1** | chr2 | + | uc021vix.1 | 70164869 | 70167775 | + |
| **MXD1** | chr2 | + | uc021viw.1 | 70157399 | 70160078 | + |
| **MAP4K3** | chr2 | - | NR_037875 | 39664556 | 39828484 | + |
| **CHMP3** | chr2 | - | NR_036454 | 86730552 | 86790620 | - |
| **TANK** | chr2 | + | uc002ubt.1 | 162101249 | 162105241 | + |
| **TANK** | chr2 | + | ENST00000436506 | 162101265 | 162103965 | + |
| **KIDINS220** | chr2 | - | ENST00000569008 | 8861263 | 8862816 | - |
| **PXDN** | chr2 | - | TCONS_00004671 | 1754992 | 1757189 | + |
| **TUBA4A** | chr2 | - | ENST00000485041 | 220117964 | 220136733 | + |
| **ZNF639** | chr3 | + | ENST00000569932 | 179058109 | 179059675 | + |
| **MYLK** | chr3 | - | NR_038266 | 123304402 | 123349666 | + |
| **GPX1** | chr3 | - | ENST00000428083 | 49402577 | 49404439 | - |
| **GHRL** | chr3 | - | NR_024145 | 10327437 | 10335133 | + |
| **GHRL** | chr3 | - | NR_004431 | 10327437 | 10335133 | + |
| **SETMAR** | chr3 | + | NR_024022 | 4345296 | 4358949 | + |
| **TMEM43** | chr3 | + | ENST00000428681 | 14186319 | 14187021 | + |
| **GSK3B** | chr3 | - | ENST00000484076 | 119813741 | 119855630 | + |
| **NUDT16** | chr3 | + | NR_033268 | 131100624 | 131107674 | + |
| **GCET2** | chr3 | - | ENST00000563632 | 111852269 | 111854206 | + |
| **ATG7** | chr3 | + | uc003bwe.1 | 11577722 | 11580151 | + |
| **C3orf26** | chr3 | + | ENST00000569034 | 99898410 | 99900574 | + |
| **OXSM** | chr3 | + | NR_026937 | 25831562 | 25836025 | + |
| **RFC4** | chr3 | - | ENST00000577781 | 186499568 | 186501968 | - |
| **TMEM44** | chr3 | - | ENST00000447139 | 194353651 | 194366130 | + |
| **NUDT16** | chr3 | + | NR_033268 | 131100624 | 131107674 | + |
| **ATG7** | chr3 | + | uc003bwe.1 | 11577722 | 11580151 | + |
| **ATG7** | chr3 | + | uc003bwe.1 | 11577722 | 11580151 | + |
| **PROK2** | chr3 | - | TCONS_00006534 | 71834485 | 71835757 | - |
| **TMEM44** | chr3 | - | ENST00000447139 | 194353651 | 194366130 | + |
| **GSK3B** | chr3 | - | ENST00000484076 | 119813741 | 119855630 | + |
| **GCET2** | chr3 | - | ENST00000563632 | 111852269 | 111854206 | + |
| **NGLY1** | chr3 | - | NR_026937 | 25831562 | 25836025 | + |
| **FXR1** | chr3 | + | NR_046073 | 180701497 | 180707562 | - |
| **FXR1** | chr3 | + | NR_033723 | 180701497 | 180707562 | - |
| **CCR2** | chr3 | + | ENST00000451485 | 46406445 | 46448550 | - |
| **TMEM44** | chr3 | - | ENST00000447139 | 194353651 | 194366130 | + |
| **ARPP21** | chr3 | + | ENST00000415706 | 35691688 | 35693453 | - |
| **PGRMC2** | chr4 | - | ENST00000505133 | 129213905 | 129387816 | + |
| **MAD2L1** | chr4 | - | ENST00000511064 | 120988112 | 121087047 | + |
| **LEF1** | chr4 | - | NR_029373 | 109093275 | 109097586 | + |
| **LEF1** | chr4 | - | NR_029374 | 109088680 | 109097586 | + |
| **LEF1** | chr4 | - | ENST00000507799 | 109093854 | 109121076 | + |
| **TET2** | chr4 | + | ENST00000504082 | 106058436 | 106061776 | - |
| **S100P** | chr4 | + | ENST00000499502 | 6689174 | 6692246 | - |
| **SCOC** | chr4 | + | uc003iie.3 | 141281970 | 141290504 | - |
| **SCOC** | chr4 | + | NR_033939 | 141204879 | 141294546 | - |
| **C4orf47** | chr4 | + | NR_028085 | 186320693 | 186347139 | - |
| **SEC31A** | chr4 | - | NR_034075 | 83814604 | 83822069 | - |
| **SEC31A** | chr4 | - | NR_034076 | 83814604 | 83822069 | - |
| **SEC31A** | chr4 | - | NR_034075 | 83814604 | 83822069 | - |
| **SEC31A** | chr4 | - | NR_034076 | 83814604 | 83822069 | - |
| **AP1AR** | chr4 | + | ENST00000562919 | 113150716 | 113152752 | - |
| **GNPDA2** | chr4 | - | NR_073094 | 44703811 | 44728651 | - |
| **PACRGL** | chr4 | + | NR_047661 | 20702035 | 20729980 | + |
| **TBC1D19** | chr4 | + | TCONS_00008015 | 26765708 | 26766256 | + |
| **SEC31A** | chr4 | - | NR_034075 | 83814604 | 83822069 | - |
| **SEC31A** | chr4 | - | NR_034076 | 83814604 | 83822069 | - |
| **HERC3** | chr4 | + | ENST00000500765 | 89630939 | 89651254 | + |
| **NFKB1** | chr4 | + | ENST00000563833 | 103421997 | 103422476 | - |
| **NFKB1** | chr4 | + | ENST00000563833 | 103421997 | 103422476 | - |
| **TICAM2** | chr5 | - | ENST00000515570 | 114938741 | 114956356 | + |
| **TICAM2** | chr5 | - | ENST00000508517 | 114937940 | 114955975 | + |
| **UGT3A2** | chr5 | - | NR_031764 | 36035118 | 36067023 | - |
| **DOCK2** | chr5 | + | ENST00000523398 | 169199969 | 169206369 | - |
| **HMMR** | chr5 | + | uc003lzi.3 | 162909609 | 162921064 | - |
| **TCF7** | chr5 | + | NR_033449 | 133451349 | 133483920 | + |
| **ARHGAP26** | chr5 | + | ENST00000432015 | 142239168 | 142248481 | - |
| **PJA2** | chr5 | - | ENST00000512693 | 108572820 | 108662070 | - |
| **HMMR** | chr5 | + | uc003lzi.3 | 162909609 | 162921064 | - |
| **HMMR** | chr5 | + | uc003lzi.3 | 162909609 | 162921064 | - |
| **HMMR** | chr5 | + | uc003lzi.3 | 162909609 | 162921064 | - |
| **TMED7-TICAM2** | chr5 | - | ENST00000515570 | 114938741 | 114956356 | + |
| **TMED7-TICAM2** | chr5 | - | ENST00000508517 | 114937940 | 114955975 | + |
| **PTCD2** | chr5 | + | BQ010989 | 71663626 | 71664322 | + |
| **CETN3** | chr5 | - | ENST00000562820 | 89684284 | 89685180 | - |
| **TMED7-TICAM2** | chr5 | - | ENST00000515570 | 114938741 | 114956356 | + |
| **TMED7-TICAM2** | chr5 | - | ENST00000508517 | 114937940 | 114955975 | + |
| **MAT2B** | chr5 | + | uc003lzi.3 | 162909609 | 162921064 | - |
| **FASTKD3** | chr5 | - | NR_036553 | 7859271 | 7869150 | - |
| **SLC12A2** | chr5 | + | uc003kun.3 | 127276131 | 127418766 | - |
| **SLC12A2** | chr5 | + | ENST00000514573 | 127302364 | 127418782 | - |
| **SLC12A2** | chr5 | + | ENST00000508353 | 127302426 | 127418573 | - |
| **SLC12A2** | chr5 | + | ENST00000501652 | 127301579 | 127418666 | - |
| **SLC12A2** | chr5 | + | ENST00000501173 | 127302240 | 127418767 | - |
| **NREP** | chr5 | - | ENST00000513221 | 111066085 | 111067878 | + |
| **VNN2** | chr6 | - | NR_034174 | 133065008 | 133079022 | - |
| **VNN2** | chr6 | - | NR_034173 | 133065008 | 133079022 | - |
| **L3MBTL3** | chr6 | + | ENST00000415964 | 130454554 | 130463972 | - |
| **CD109** | chr6 | + | uc003pho.1 | 74403625 | 74405743 | - |
| **USP49** | chr6 | - | uc003orh.1 | 41757633 | 41760146 | - |
| **HLA-DRB1** | chr6 | - | uc010jub.1 | 32546122 | 32546497 | - |
| **BTN3A1** | chr6 | + | uc011dkm.2 | 26422316 | 26431928 | + |
| **NOL7** | chr6 | + | ENST00000566170 | 13614342 | 13615387 | - |
| **EEF1E1** | chr6 | - | NR_037618 | 8013799 | 8102828 | - |
| **ACAT2** | chr6 | + | NR_037166 | 160181290 | 160183364 | - |
| **MICA** | chr6 | + | NR_036523 | 31367560 | 31383036 | + |
| **BTN3A1** | chr6 | + | uc011dkm.2 | 26422316 | 26431928 | + |
| **PSMG4** | chr6 | + | uc003mvi.1 | 3229005 | 3288894 | + |
| **VNN2** | chr6 | - | NR_034174 | 133065008 | 133079022 | - |
| **VNN2** | chr6 | - | NR_034173 | 133065008 | 133079022 | - |
| **RNF8** | chr6 | + | NR_046399 | 37321747 | 37362514 | + |
| **CD109** | chr6 | + | uc003pho.1 | 74403625 | 74405743 | - |
| **CD109** | chr6 | + | uc003pho.1 | 74403625 | 74405743 | - |
| **CNPY3** | chr6 | + | TCONS_00012717 | 42909007 | 42915981 | - |
| **SAMD3** | chr6 | - | ENST00000415964 | 130454554 | 130463972 | - |
| **STXBP5** | chr6 | + | ENST00000367477 | 147164249 | 147525750 | - |
| **STXBP5** | chr6 | + | NR_034115 | 147162524 | 147525750 | - |
| **FBXO5** | chr6 | - | ENST00000442269 | 153304884 | 153310358 | + |
| **QKI** | chr6 | + | ENST00000446476 | 164007614 | 164009197 | + |
| **QKI** | chr6 | + | ENST00000446476 | 164007614 | 164009197 | + |
| **NQO2** | chr6 | + | ENST00000563388 | 3025024 | 3027659 | + |
| **NQO2** | chr6 | + | NR_026856 | 2988200 | 2991405 | + |
| **STXBP5** | chr6 | + | ENST00000367477 | 147164249 | 147525750 | - |
| **STXBP5** | chr6 | + | NR_034115 | 147162524 | 147525750 | - |
| **QKI** | chr6 | + | ENST00000446476 | 164007614 | 164009197 | + |
| **AHI1** | chr6 | - | uc003qgn.3 | 135818938 | 136037193 | + |
| **AHI1** | chr6 | - | ENST00000444302 | 135622705 | 135628296 | + |
| **AHI1** | chr6 | - | uc003qgo.3 | 135818938 | 136037193 | + |
| **BLOC1S5** | chr6 | - | NR_037618 | 8013799 | 8102828 | - |
| **TNFAIP3** | chr6 | + | ENST00000431144 | 138178836 | 138182628 | - |
| **TNFAIP3** | chr6 | + | uc003qhq.1 | 138144811 | 138189370 | - |
| **MAPK13** | chr6 | + | ENST00000526611 | 36114474 | 36164980 | - |
| **HILPDA** | chr7 | + | ENST00000478818 | 128106616 | 128109225 | + |
| **TRIM73** | chr7 | + | ENST00000404291 | 74995622 | 75024657 | + |
| **TRIM73** | chr7 | + | ENST00000416371 | 75022638 | 75024555 | - |
| **FIGNL1** | chr7 | - | AL137474 | 50518333 | 50521015 | + |
| **TBXAS1** | chr7 | + | NR_029394 | 139528951 | 139720125 | + |
| **AHR** | chr7 | + | ENST00000452249 | 17325902 | 17338070 | - |
| **AHR** | chr7 | + | ENST00000433005 | 17325898 | 17338981 | - |
| **GATS** | chr7 | - | NR_028040 | 99798277 | 99869855 | - |
| **DNAJC30** | chr7 | - | NR_037776 | 73097897 | 73112551 | + |
| **TBXAS1** | chr7 | + | NR_029394 | 139528951 | 139720125 | + |
| **LSM5** | chr7 | - | NR_024466 | 32524944 | 32530475 | - |
| **TES** | chr7 | + | uc003vhq.1 | 115893169 | 115895118 | + |
| **STEAP4** | chr7 | - | ENST00000434733 | 87906315 | 87921781 | + |
| **FBXL13** | chr7 | - | ENST00000420058 | 102613968 | 102629303 | + |
| **TES** | chr7 | + | uc003vhq.1 | 115893169 | 115895118 | + |
| **H2AFV** | chr7 | - | ENST00000443162 | 44888014 | 44889167 | + |
| **SNX10** | chr7 | + | ENST00000451368 | 26411763 | 26415892 | - |
| **SNX10** | chr7 | + | ENST00000451264 | 26411951 | 26416321 | - |
| **ZYX** | chr7 | + | ENST00000429630 | 143076784 | 143077588 | - |
| **FBXL13** | chr7 | - | ENST00000420058 | 102613968 | 102629303 | + |
| **GGCT** | chr7 | - | NR_037669 | 30536236 | 30544457 | - |
| **CDK6** | chr7 | - | ENST00000435695 | 92465796 | 92546501 | + |
| **CDK6** | chr7 | - | ENST00000452050 | 92465802 | 92546437 | + |
| **DUS4L** | chr7 | + | NR_073004 | 107204401 | 107218968 | + |
| **DUS4L** | chr7 | + | NR_073005 | 107204401 | 107218968 | + |
| **TBXAS1** | chr7 | + | NR_029394 | 139528951 | 139720125 | + |
| **H2AFV** | chr7 | - | ENST00000443162 | 44888014 | 44889167 | + |
| **BNIP3L** | chr8 | + | uc003xew.3 | 26236776 | 26240469 | - |
| **VPS13B** | chr8 | + | ENST00000521696 | 100008990 | 100025272 | - |
| **E2F5** | chr8 | + | ENST00000562577 | 86084311 | 86089276 | - |
| **FABP5** | chr8 | + | ENST00000518880 | 82192105 | 82193681 | - |
| **LYN** | chr8 | + | ENST00000518552 | 56806153 | 56808276 | + |
| **LYN** | chr8 | + | ENST00000518552 | 56806153 | 56808276 | + |
| **TOX** | chr8 | - | ENST00000518993 | 60031776 | 60033905 | + |
| **TOX** | chr8 | - | ENST00000523683 | 60031598 | 60032590 | + |
| **TOX** | chr8 | - | ENST00000517898 | 60032504 | 60033874 | + |
| **DUSP26** | chr8 | - | ENST00000523063 | 33462373 | 33896003 | + |
| **BAALC** | chr8 | + | ENST00000523775 | 104240652 | 104241542 | - |
| **DPY19L4** | chr8 | + | ENST00000518613 | 95804510 | 95806064 | - |
| **DPY19L4** | chr8 | + | ENST00000510185 | 95804459 | 95805334 | - |
| **DPY19L4** | chr8 | + | ENST00000521706 | 95803870 | 95805191 | - |
| **VPS13B** | chr8 | + | ENST00000521696 | 100008990 | 100025272 | - |
| **VPS13B** | chr8 | + | ENST00000521696 | 100008990 | 100025272 | - |
| **FGFR1** | chr8 | - | ENST00000533301 | 38258053 | 38259201 | - |
| **BAALC** | chr8 | + | ENST00000523775 | 104240652 | 104241542 | - |
| **OTUD6B** | chr8 | + | ENST00000522817 | 92072136 | 92082351 | - |
| **RRM2B** | chr8 | - | ENST00000520820 | 103251621 | 103264402 | + |
| **RGS3** | chr9 | + | ENST00000428429 | 116332469 | 116352299 | - |
| **WDR34** | chr9 | - | ENST00000428643 | 131425412 | 131425857 | - |
| **CAMSAP1** | chr9 | - | ENST00000423793 | 138799657 | 138805359 | + |
| **NIPSNAP3B** | chr9 | + | NR_024011 | 107536632 | 107540045 | + |
| **ABCA1** | chr9 | - | NR_024011 | 107536632 | 107540045 | + |
| **UGCG** | chr9 | + | ENST00000366375 | 114680536 | 114681204 | - |
| **CDKN2A** | chr9 | - | NR_047537 | 21994789 | 22121093 | + |
| **CDKN2A** | chr9 | - | NR_047538 | 21994789 | 22121093 | + |
| **CDKN2A** | chr9 | - | NR_047541 | 21994789 | 22077889 | + |
| **CDKN2A** | chr9 | - | NR_047536 | 21994789 | 22121093 | + |
| **CDKN2A** | chr9 | - | NR_047542 | 21994789 | 22077889 | + |
| **CDKN2A** | chr9 | - | NR_047543 | 21994789 | 22121093 | + |
| **UHRF2** | chr9 | + | NR_046386 | 6413150 | 6507051 | + |
| **HSDL2** | chr9 | + | uc004bgd.3 | 115178826 | 115180063 | + |
| **DNAJC9** | chr10 | - | ENST00000453189 | 74998539 | 75003156 | - |
| **DNAJC9** | chr10 | - | ENST00000457758 | 75012789 | 75014102 | + |
| **DNAJC9** | chr10 | - | uc001jtu.1 | 75012548 | 75014101 | + |
| **PDCD4** | chr10 | + | ENST00000420367 | 112629625 | 112631991 | - |
| **PDCD4** | chr10 | + | NR_026932 | 112628647 | 112630662 | - |
| **SRGN** | chr10 | + | uc001jpa.1 | 70847867 | 70848929 | + |
| **SRGN** | chr10 | + | NR_036430 | 70847827 | 70864567 | + |
| **NCOA4** | chr10 | + | NR_073029 | 51592080 | 51623386 | - |
| **MRPS16** | chr10 | - | ENST00000453189 | 74998539 | 75003156 | - |
| **MRPS16** | chr10 | - | ENST00000457758 | 75012789 | 75014102 | + |
| **MRPS16** | chr10 | - | uc001jtu.1 | 75012548 | 75014101 | + |
| **PARD3** | chr10 | - | ENST00000446211 | 35104694 | 35105314 | + |
| **PARD3** | chr10 | - | ENST00000446211 | 35104694 | 35105314 | + |
| **NSUN6** | chr10 | - | ENST00000436485 | 18830514 | 18833316 | - |
| **NSUN6** | chr10 | - | uc001iqb.3 | 18802045 | 18834577 | - |
| **NSUN6** | chr10 | - | ENST00000449529 | 18943479 | 18948185 | - |
| **NSUN6** | chr10 | - | ENST00000444660 | 18943616 | 18948196 | - |
| **KLF6** | chr10 | - | NR_027653 | 3818187 | 3827473 | - |
| **KLF6** | chr10 | - | NR_027653 | 3818187 | 3827473 | - |
| **CELF2** | chr10 | + | uc001ikr.1 | 11295753 | 11298627 | + |
| **ACBD5** | chr10 | - | ENST00000574842 | 27532058 | 27539733 | + |
| **ANKRD16** | chr10 | - | uc001iio.1 | 5903871 | 5906246 | - |
| **HSPA14** | chr10 | + | NR_034181 | 14920781 | 14946304 | + |
| **SUV39H2** | chr10 | + | NR_034181 | 14920781 | 14946304 | + |
| **PARD3** | chr10 | - | ENST00000446211 | 35104694 | 35105314 | + |
| **PARD3** | chr10 | - | ENST00000446211 | 35104694 | 35105314 | + |
| **NCOA4** | chr10 | + | NR_073029 | 51592080 | 51623386 | - |
| **ENTPD1** | chr10 | + | ENST00000452942 | 97635156 | 97667639 | - |
| **ENTPD1** | chr10 | + | ENST00000452942 | 97635156 | 97667639 | - |
| **BLNK** | chr10 | - | NR_047682 | 97951454 | 98031273 | - |
| **BLNK** | chr10 | - | NR_047680 | 97951454 | 98031273 | - |
| **GOT1** | chr10 | - | ENST00000416191 | 101190947 | 101195542 | + |
| **NSMCE4A** | chr10 | - | ENST00000437593 | 123687826 | 123711480 | + |
| **FAS** | chr10 | + | ENST00000562983 | 90775592 | 90776816 | + |
| **FAS** | chr10 | + | NR_028034 | 90750287 | 90775542 | + |
| **FAS** | chr10 | + | NR_028033 | 90750287 | 90775542 | + |
| **PARD3** | chr10 | - | ENST00000446211 | 35104694 | 35105314 | + |
| **PARD3** | chr10 | - | ENST00000446211 | 35104694 | 35105314 | + |
| **PARD3** | chr10 | - | ENST00000446211 | 35104694 | 35105314 | + |
| **PARD3** | chr10 | - | ENST00000446211 | 35104694 | 35105314 | + |
| **CREM** | chr10 | + | ENST00000457255 | 35389817 | 35415948 | - |
| **FAS** | chr10 | + | ENST00000562983 | 90775592 | 90776816 | + |
| **FAS** | chr10 | + | NR_028034 | 90750287 | 90775542 | + |
| **FAS** | chr10 | + | NR_028033 | 90750287 | 90775542 | + |
| **SUV39H2** | chr10 | + | NR_034181 | 14920781 | 14946304 | + |
| **TIMM23** | chr10 | - | NR_073029 | 51592080 | 51623386 | - |
| **HELLS** | chr10 | + | ENST00000432120 | 96337195 | 96370995 | + |
| **ZW10** | chr11 | - | ENST00000543486 | 113641114 | 113642413 | - |
| **CASP12** | chr11 | - | ENST00000532510 | 104772275 | 104779432 | - |
| **CASP12** | chr11 | - | ENST00000534659 | 104772276 | 104774437 | - |
| **CASP12** | chr11 | - | ENST00000528437 | 104772275 | 104788872 | - |
| **CASP12** | chr11 | - | ENST00000527617 | 104777284 | 104788874 | - |
| **NARS2** | chr11 | - | ENST00000513207 | 78135027 | 78140882 | - |
| **STARD10** | chr11 | - | TCONS_00019700 | 72514716 | 72524260 | - |
| **TCIRG1** | chr11 | + | ENST00000529934 | 67818206 | 67820852 | + |
| **TCIRG1** | chr11 | + | ENST00000526897 | 67819559 | 67821161 | + |
| **HBG2** | chr11 | - | ENST00000433329 | 5263349 | 5264767 | - |
| **HBG2** | chr11 | - | ENST00000454892 | 5263183 | 5264496 | - |
| **SESN3** | chr11 | - | ENST00000543573 | 94965734 | 94967268 | + |
| **KIF18A** | chr11 | - | ENST00000525309 | 28000215 | 28041122 | - |
| **HBG1** | chr11 | - | ENST00000433329 | 5263349 | 5264767 | - |
| **HBG1** | chr11 | - | ENST00000454892 | 5263183 | 5264496 | - |
| **APLP2** | chr11 | + | NR_024515 | 129939715 | 130014706 | + |
| **RTN3** | chr11 | + | ENST00000546282 | 63534955 | 63536113 | - |
| **RTN3** | chr11 | + | NR_049751 | 63448921 | 63527363 | + |
| **LGALS12** | chr11 | + | TCONS_00019317 | 63261874 | 63268075 | + |
| **CHEK1** | chr11 | + | NR_045205 | 125496123 | 125527042 | + |
| **APLP2** | chr11 | + | NR_024515 | 129939715 | 130014706 | + |
| **CD44** | chr11 | + | ENST00000510619 | 35234096 | 35235554 | + |
| **CD44** | chr11 | + | ENST00000510619 | 35234096 | 35235554 | + |
| **FAM111B** | chr11 | + | ENST00000532845 | 58903397 | 58907588 | - |
| **C11orf67** | chr11 | + | ENST00000525594 | 77540699 | 77583308 | - |
| **C11orf67** | chr11 | + | ENST00000527012 | 77561649 | 77562557 | + |
| **RTN3** | chr11 | + | ENST00000546282 | 63534955 | 63536113 | - |
| **RTN3** | chr11 | + | NR_049751 | 63448921 | 63527363 | + |
| **TMEM25** | chr11 | + | ENST00000532597 | 118382625 | 118401292 | - |
| **APLP2** | chr11 | + | NR_024515 | 129939715 | 130014706 | + |
| **FAM111B** | chr11 | + | ENST00000532845 | 58903397 | 58907588 | - |
| **LGALS12** | chr11 | + | TCONS_00019317 | 63261874 | 63268075 | + |
| **RTN3** | chr11 | + | ENST00000546282 | 63534955 | 63536113 | - |
| **RTN3** | chr11 | + | NR_049751 | 63448921 | 63527363 | + |
| **PHF21A** | chr11 | - | ENST00000528480 | 46138128 | 46138869 | + |
| **HRAS** | chr11 | - | ENST00000526431 | 528906 | 529659 | + |
| **CD44** | chr11 | + | ENST00000510619 | 35234096 | 35235554 | + |
| **CD44** | chr11 | + | ENST00000510619 | 35234096 | 35235554 | + |
| **APLP2** | chr11 | + | NR_024515 | 129939715 | 130014706 | + |
| **CD44** | chr11 | + | ENST00000510619 | 35234096 | 35235554 | + |
| **PHF21A** | chr11 | - | ENST00000528480 | 46138128 | 46138869 | + |
| **SBF2** | chr11 | - | uc001mie.3 | 9801925 | 9812791 | + |
| **SBF2** | chr11 | - | ENST00000526617 | 9806697 | 9812784 | + |
| **LGALS12** | chr11 | + | TCONS_00019317 | 63261874 | 63268075 | + |
| **SLC15A3** | chr11 | - | NR_027391 | 60704554 | 60719257 | - |
| **TMPO** | chr12 | + | NR_027157 | 98906750 | 98910004 | - |
| **TMPO** | chr12 | + | ENST00000546421 | 98909071 | 98909988 | - |
| **LTA4H** | chr12 | - | ENST00000551849 | 96390298 | 96405267 | + |
| **BTG1** | chr12 | - | ENST00000501008 | 92539348 | 92561336 | + |
| **BTG1** | chr12 | - | NR_046159 | 92378751 | 92535489 | - |
| **OS9** | chr12 | + | ENST00000549477 | 58087914 | 58115293 | - |
| **OS9** | chr12 | + | ENST00000549477 | 58087914 | 58115293 | - |
| **PCED1B** | chr12 | + | ENST00000547748 | 47631516 | 47672804 | - |
| **PCED1B** | chr12 | + | NR_026544 | 47602202 | 47610226 | - |
| **PCED1B** | chr12 | + | uc001rpo.1 | 47599681 | 47601754 | - |
| **PCED1B** | chr12 | + | ENST00000550426 | 47599680 | 47609983 | - |
| **PCED1B** | chr12 | + | ENST00000552990 | 47599683 | 47610227 | - |
| **KLRC4** | chr12 | - | ENST00000500682 | 10516367 | 10551105 | + |
| **WDR66** | chr12 | + | ENST00000538710 | 122445339 | 122457920 | - |
| **HCAR3** | chr12 | - | ENST00000545293 | 123198837 | 123200255 | + |
| **TESC** | chr12 | - | ENST00000547006 | 117537285 | 117579896 | + |
| **TMPO** | chr12 | + | NR_027157 | 98906750 | 98910004 | - |
| **TMPO** | chr12 | + | ENST00000546421 | 98909071 | 98909988 | - |
| **TESC** | chr12 | - | ENST00000547006 | 117537285 | 117579896 | + |
| **VEZT** | chr12 | + | NR_038242 | 95611521 | 95696566 | + |
| **TMPO** | chr12 | + | NR_027157 | 98906750 | 98910004 | - |
| **TMPO** | chr12 | + | ENST00000546421 | 98909071 | 98909988 | - |
| **OS9** | chr12 | + | ENST00000549477 | 58087914 | 58115293 | - |
| **SELPLG** | chr12 | - | ENST00000547282 | 109029624 | 109034693 | + |
| **TMEM194A** | chr12 | - | TCONS_00020805 | 57477094 | 57481617 | - |
| **PLEKHA5** | chr12 | + | ENST00000501211 | 19300007 | 19307593 | + |
| **CCDC41** | chr12 | - | NR_027035 | 94853778 | 94856344 | + |
| **MAGOHB** | chr12 | - | uc010shg.2 | 10741076 | 10752434 | - |
| **MAGOHB** | chr12 | - | ENST00000503499 | 10741984 | 10750808 | - |
| **MAGOHB** | chr12 | - | ENST00000510134 | 10741381 | 10752268 | - |
| **MAGOHB** | chr12 | - | uc009zhn.3 | 10741076 | 10752434 | - |
| **MAGOHB** | chr12 | - | ENST00000535939 | 10741771 | 10752164 | - |
| **MAGOHB** | chr12 | - | uc009zho.3 | 10741076 | 10752434 | - |
| **OS9** | chr12 | + | ENST00000549477 | 58087914 | 58115293 | - |
| **STAT6** | chr12 | - | TCONS_00020805 | 57477094 | 57481617 | - |
| **NAB2** | chr12 | + | TCONS_00020805 | 57477094 | 57481617 | - |
| **PLEKHA5** | chr12 | + | ENST00000501211 | 19300007 | 19307593 | + |
| **C12orf23** | chr12 | + | ENST00000570282 | 107347806 | 107349275 | - |
| **SLC15A4** | chr12 | - | uc001uhw.3 | 129297748 | 129299068 | - |
| **LYZ** | chr12 | + | ENST00000548626 | 69746948 | 69747436 | - |
| **RNASEH2B** | chr13 | + | NR_046552 | 51456514 | 51484848 | - |
| **CENPJ** | chr13 | - | NR_047594 | 25456411 | 25497027 | - |
| **CENPJ** | chr13 | - | BC042735 | 25497105 | 25498565 | + |
| **KATNAL1** | chr13 | - | ENST00000412722 | 30890496 | 30894040 | - |
| **GPR65** | chr14 | + | ENST00000554433 | 88477130 | 88481955 | - |
| **GLRX5** | chr14 | + | NR_001459 | 95999248 | 96001209 | - |
| **GLRX5** | chr14 | + | TCONS_00022845 | 95988348 | 95992377 | - |
| **GLRX5** | chr14 | + | ENST00000500370 | 95998633 | 96001137 | - |
| **GLRX5** | chr14 | + | ENST00000555866 | 95999250 | 96000923 | - |
| **ARHGAP5** | chr14 | + | NR_027263 | 32544624 | 32545905 | - |
| **NUMB** | chr14 | - | ENST00000556578 | 73738889 | 73740789 | - |
| **TCL1A** | chr14 | - | ENST00000547644 | 96178083 | 96222004 | + |
| **TCL1A** | chr14 | - | NR_049726 | 96176303 | 96180533 | - |
| **FUT8** | chr14 | + | NR_024334 | 65877310 | 65879335 | - |
| **NUMB** | chr14 | - | ENST00000556578 | 73738889 | 73740789 | - |
| **NUMB** | chr14 | - | ENST00000556578 | 73738889 | 73740789 | - |
| **LRR1** | chr14 | + | NR_037793 | 50065414 | 50081390 | + |
| **IFT43** | chr14 | + | NR_045664 | 76452095 | 76550416 | + |
| **LRR1** | chr14 | + | NR_037793 | 50065414 | 50081390 | + |
| **SLC7A7** | chr14 | - | ENST00000554194 | 23175423 | 23235756 | - |
| **ACTN1** | chr14 | - | ENST00000553944 | 69446757 | 69448270 | + |
| **CCPG1** | chr15 | - | NR_037923 | 55647420 | 55790782 | - |
| **GABPB1** | chr15 | - | NR_026891 | 50641134 | 50647076 | - |
| **GABPB1** | chr15 | - | ENST00000561289 | 50647741 | 50648611 | + |
| **GABPB1** | chr15 | - | ENST00000560359 | 50647524 | 50648567 | + |
| **GABPB1** | chr15 | - | uc001zyi.3 | 50647663 | 50650503 | + |
| **GABPB1** | chr15 | - | ENST00000499326 | 50647749 | 50648633 | + |
| **OIP5** | chr15 | - | TCONS_00024099 | 41590238 | 41598727 | - |
| **OIP5** | chr15 | - | NR_026757 | 41576200 | 41591795 | + |
| **SCG5** | chr15 | + | uc001zhb.2 | 32962079 | 32963900 | + |
| **IGF1R** | chr15 | + | uc021sxi.1 | 99441844 | 99443609 | + |
| **IGF1R** | chr15 | + | ENST00000560221 | 99190179 | 99190600 | - |
| **ZSCAN2** | chr15 | + | ENST00000427525 | 85174709 | 85178652 | + |
| **ST20** | chr15 | - | NR_037654 | 80135888 | 80189339 | - |
| **LEO1** | chr15 | - | ENST00000557871 | 52211269 | 52222824 | - |
| **TCF12** | chr15 | + | NR_015419 | 57178367 | 57210697 | - |
| **SPINT1** | chr15 | + | ENST00000568525 | 41130536 | 41136498 | - |
| **TSPAN3** | chr15 | - | ENST00000569742 | 77334177 | 77335533 | - |
| **TBC1D2B** | chr15 | - | NR_026998 | 78285574 | 78286567 | - |
| **PRC1** | chr15 | - | uc021suk.1 | 91504656 | 91506348 | + |
| **HAUS2** | chr15 | + | ENST00000567089 | 42861495 | 42862192 | - |
| **PRC1** | chr15 | - | uc021suk.1 | 91504656 | 91506348 | + |
| **PRC1** | chr15 | - | uc021suk.1 | 91504656 | 91506348 | + |
| **GABPB1** | chr15 | - | NR_026891 | 50641134 | 50647076 | - |
| **GABPB1** | chr15 | - | ENST00000561289 | 50647741 | 50648611 | + |
| **GABPB1** | chr15 | - | ENST00000560359 | 50647524 | 50648567 | + |
| **GABPB1** | chr15 | - | uc001zyi.3 | 50647663 | 50650503 | + |
| **GABPB1** | chr15 | - | ENST00000499326 | 50647749 | 50648633 | + |
| **CATSPER2** | chr15 | - | uc021ska.1 | 43925928 | 43927115 | - |
| **DYX1C1** | chr15 | - | NR_037923 | 55647420 | 55790782 | - |
| **TSPAN3** | chr15 | - | ENST00000569742 | 77334177 | 77335533 | - |
| **AP3S2** | chr15 | - | NR_037582 | 90373830 | 90437617 | - |
| **CHP1** | chr15 | + | NR_026757 | 41576200 | 41591795 | + |
| **CHP1** | chr15 | + | ENST00000560545 | 41576207 | 41577887 | + |
| **DYX1C1** | chr15 | - | NR_037923 | 55647420 | 55790782 | - |
| **ADPGK** | chr15 | - | NR_023319 | 73043707 | 73076126 | - |
| **ADPGK** | chr15 | - | NR_023318 | 73043707 | 73076126 | - |
| **TMOD3** | chr15 | + | ENST00000557871 | 52211269 | 52222824 | - |
| **TBC1D2B** | chr15 | - | NR_026998 | 78285574 | 78286567 | - |
| **CATSPER2** | chr15 | - | uc021ska.1 | 43925928 | 43927115 | - |
| **HDGFRP3** | chr15 | - | TCONS_00023269 | 83876654 | 83896949 | + |
| **TSPAN3** | chr15 | - | ENST00000569742 | 77334177 | 77335533 | - |
| **TCF12** | chr15 | + | NR_015419 | 57178367 | 57210697 | - |
| **HAUS2** | chr15 | + | ENST00000567089 | 42861495 | 42862192 | - |
| **CYBA** | chr16 | - | uc002flc.1 | 88709698 | 88712089 | - |
| **LPCAT2** | chr16 | + | ENST00000576365 | 55572111 | 55575939 | + |
| **YPEL3** | chr16 | - | ENST00000569011 | 30103638 | 30104110 | + |
| **TPSD1** | chr16 | + | ENST00000568091 | 1310952 | 1313846 | - |
| **CMIP** | chr16 | + | NR_045112 | 81698958 | 81700879 | + |
| **C16orf72** | chr16 | + | ENST00000574285 | 9198704 | 9200618 | + |
| **ZG16B** | chr16 | + | NR_073012 | 2867163 | 2871723 | + |
| **CORO1A** | chr16 | + | NR_037608 | 30205753 | 30215650 | + |
| **PRSS21** | chr16 | + | NR_073012 | 2867163 | 2871723 | + |
| **PRSS21** | chr16 | + | NR_073012 | 2867163 | 2871723 | + |
| **CLUAP1** | chr16 | + | ENST00000573820 | 3594430 | 3595785 | + |
| **IL32** | chr16 | + | ENST00000573130 | 3103367 | 3106233 | - |
| **CLUAP1** | chr16 | + | ENST00000573820 | 3594430 | 3595785 | + |
| **ORC6** | chr16 | + | NR_037620 | 46723557 | 46732306 | + |
| **PRSS21** | chr16 | + | NR_073012 | 2867163 | 2871723 | + |
| **SKA2** | chr17 | - | uc002ixb.3 | 57183958 | 57195629 | + |
| **TRIM37** | chr17 | - | uc002ixb.3 | 57183958 | 57195629 | + |
| **COX11** | chr17 | - | NR_027942 | 53029258 | 53046064 | - |
| **COX11** | chr17 | - | NR_027941 | 53029258 | 53046064 | - |
| **PIGL** | chr17 | + | ENST00000431149 | 16229798 | 16250988 | + |
| **SCIMP** | chr17 | - | NR_034082 | 5095378 | 5138931 | + |
| **NFE2L1** | chr17 | + | ENST00000578660 | 46122502 | 46125412 | - |
| **HS3ST3B1** | chr17 | + | ENST00000584683 | 14230651 | 14232791 | + |
| **RPAIN** | chr17 | + | NR_027682 | 5322960 | 5336340 | + |
| **ALYREF** | chr17 | - | ENST00000579981 | 79836542 | 79837433 | + |
| **ALYREF** | chr17 | - | ENST00000582866 | 79836300 | 79838982 | - |
| **CD300E** | chr17 | - | ENST00000569279 | 72599875 | 72603368 | + |
| **CD300E** | chr17 | - | ENST00000577560 | 72602645 | 72603330 | + |
| **ALDOC** | chr17 | - | ENST00000585189 | 26900134 | 26901261 | + |
| **RPAIN** | chr17 | + | NR_027682 | 5322960 | 5336340 | + |
| **BRCA1** | chr17 | - | NR_027676 | 41196311 | 41277340 | - |
| **SKA2** | chr17 | - | uc002ixb.3 | 57183958 | 57195629 | + |
| **RNASEK** | chr17 | + | NR_037715 | 6915735 | 6917852 | + |
| **RNASEK** | chr17 | + | NR_037717 | 6915735 | 6920843 | + |
| **PSMC3IP** | chr17 | - | NR_045670 | 40724327 | 40729849 | - |
| **CD300LB** | chr17 | - | TCONS_00026160 | 72530813 | 72534060 | - |
| **RPAIN** | chr17 | + | NR_027682 | 5322960 | 5336340 | + |
| **RPAIN** | chr17 | + | NR_027682 | 5322960 | 5336340 | + |
| **CNTROB** | chr17 | + | BE671281 | 7855397 | 7859886 | + |
| **TMEM235** | chr17 | + | uc021uec.1 | 76220156 | 76220780 | - |
| **BRCA1** | chr17 | - | NR_027676 | 41196311 | 41277340 | - |
| **BIRC5** | chr17 | + | uc021uec.1 | 76220156 | 76220780 | - |
| **ARRB2** | chr17 | + | ENST00000497885 | 4607524 | 4608824 | + |
| **ARRB2** | chr17 | + | NR_047516 | 4613788 | 4624795 | + |
| **RPAIN** | chr17 | + | NR_027682 | 5322960 | 5336340 | + |
| **SUZ12** | chr17 | + | ENST00000583346 | 30330221 | 30333707 | - |
| **SPAG5** | chr17 | - | ENST00000585189 | 26900134 | 26901261 | + |
| **LRRC37A3** | chr17 | - | ENST00000577938 | 62888846 | 62892521 | + |
| **LRRC37A3** | chr17 | - | ENST00000584959 | 62888941 | 62906298 | + |
| **BRCA1** | chr17 | - | NR_027676 | 41196311 | 41277340 | - |
| **BIRC5** | chr17 | + | uc021uec.1 | 76220156 | 76220780 | - |
| **CENPV** | chr17 | - | ENST00000431149 | 16229798 | 16250988 | + |
| **NT5C3L** | chr17 | - | NR_033465 | 39981333 | 39992523 | - |
| **NT5C3L** | chr17 | - | NR_033464 | 39981333 | 39992488 | - |
| **ARRB2** | chr17 | + | ENST00000497885 | 4607524 | 4608824 | + |
| **ARRB2** | chr17 | + | NR_047516 | 4613788 | 4624795 | + |
| **TTC19** | chr17 | + | NR_037985 | 15902693 | 15932723 | + |
| **PSMC3IP** | chr17 | - | NR_045670 | 40724327 | 40729849 | - |
| **ZNF232** | chr17 | - | ENST00000570712 | 5017067 | 5017671 | + |
| **ZNF232** | chr17 | - | ENST00000413077 | 5015226 | 5017672 | + |
| **COPZ2** | chr17 | - | ENST00000578660 | 46122502 | 46125412 | - |
| **SNRPD1** | chr18 | + | ENST00000577906 | 19210098 | 19213485 | + |
| **C18orf1** | chr18 | + | uc002ksd.1 | 13457207 | 13459158 | + |
| **KDSR** | chr18 | - | AW028711 | 61034977 | 61048862 | + |
| **C18orf1** | chr18 | + | uc002ksd.1 | 13457207 | 13459158 | + |
| **NETO1** | chr18 | - | ENST00000580564 | 70535622 | 70548634 | + |
| **SLC39A6** | chr18 | - | NR_040110 | 33709836 | 33754684 | + |
| **THOC1** | chr18 | - | ENST00000581677 | 268147 | 270278 | + |
| **ZNF544** | chr19 | + | TCONS_00027642 | 58782948 | 58785962 | + |
| **B3GNT8** | chr19 | - | uc002oqt.1 | 41931315 | 41933463 | - |
| **ZNF440** | chr19 | + | uc021upk.1 | 11943741 | 11946424 | + |
| **ZNF8** | chr19 | + | TCONS_00027642 | 58782948 | 58785962 | + |
| **ZNF8** | chr19 | + | TCONS_00027643 | 58787664 | 58788877 | + |
| **ZNF470** | chr19 | + | uc002qnk.1 | 57056934 | 57078780 | - |
| **ZNF583** | chr19 | + | NR_037161 | 56905044 | 56910539 | + |
| **ZNF583** | chr19 | + | NR_037159 | 56905044 | 56910539 | + |
| **ATP5SL** | chr19 | - | uc002oqt.1 | 41931315 | 41933463 | - |
| **ZNF91** | chr19 | - | uc002nrh.1 | 23586007 | 23598873 | - |
| **ZNF528** | chr19 | + | uc002pzg.1 | 52897579 | 52901010 | - |
| **ZNF540** | chr19 | + | NR_038249 | 38042272 | 38078248 | + |
| **ZNF540** | chr19 | + | NR_038249 | 38042272 | 38078248 | + |
| **ZFP28** | chr19 | + | uc002qnk.1 | 57056934 | 57078780 | - |
| **ATP5SL** | chr19 | - | uc002oqt.1 | 41931315 | 41933463 | - |
| **ZNF432** | chr19 | - | ENST00000569091 | 52561742 | 52566956 | - |
| **TPD52L2** | chr20 | + | NR_045370 | 62507483 | 62512243 | + |
| **TPD52L2** | chr20 | + | NR_045090 | 62496580 | 62522898 | + |
| **NDUFAF5** | chr20 | + | NR_029377 | 13765671 | 13799067 | + |
| **TPD52L2** | chr20 | + | NR_045370 | 62507483 | 62512243 | + |
| **TPD52L2** | chr20 | + | NR_045090 | 62496580 | 62522898 | + |
| **FAM83D** | chr20 | + | ENST00000570096 | 37590567 | 37590754 | - |
| **PCNA** | chr20 | - | NR_028370 | 5100231 | 5100615 | + |
| **NDUFAF5** | chr20 | + | NR_029377 | 13765671 | 13799067 | + |
| **ITCH** | chr20 | + | ENST00000418598 | 33038734 | 33042357 | + |
| **NSFL1C** | chr20 | - | AW235165 | 1416447 | 1416866 | + |
| **TPD52L2** | chr20 | + | NR_045370 | 62507483 | 62512243 | + |
| **TPD52L2** | chr20 | + | NR_045090 | 62496580 | 62522898 | + |
| **C20orf24** | chr20 | + | NR_026562 | 35234136 | 35240960 | + |
| **C20orf24** | chr20 | + | NR_026562 | 35234136 | 35240960 | + |
| **NOP56** | chr20 | + | NR_027700 | 2633177 | 2639039 | + |
| **TPD52L2** | chr20 | + | NR_045370 | 62507483 | 62512243 | + |
| **TPD52L2** | chr20 | + | NR_045090 | 62496580 | 62522898 | + |
| **TPD52L2** | chr20 | + | NR_045370 | 62507483 | 62512243 | + |
| **TPD52L2** | chr20 | + | NR_045090 | 62496580 | 62522898 | + |
| **ESF1** | chr20 | - | NR_029377 | 13765671 | 13799067 | + |
| **KCNJ15** | chr21 | + | uc002ywy.3 | 39645397 | 39647443 | + |
| **IFNAR2** | chr21 | + | NR_038974 | 34637936 | 34638565 | - |
| **ATP5J** | chr21 | - | NR_072999 | 27011593 | 27089874 | + |
| **PCNT** | chr21 | + | uc021wka.1 | 47740936 | 47744424 | - |
| **PSMG1** | chr21 | - | NR_049728 | 40547371 | 40555440 | - |
| **BRWD1** | chr21 | - | NR_049728 | 40547371 | 40555440 | - |
| **PSMG1** | chr21 | - | NR_049728 | 40547371 | 40555440 | - |
| **WDR4** | chr21 | - | TCONS_00029300 | 44255638 | 44257805 | - |
| **MIS18A** | chr21 | - | ENST00000453549 | 33650173 | 33653299 | + |
| **BACH1** | chr21 | + | NR_027655 | 30677559 | 30734217 | + |
| **XBP1** | chr22 | - | ENST00000458080 | 29196670 | 29244547 | + |
| **IGLL1** | chr22 | - | ENST00000458318 | 23909250 | 23915694 | + |
| **GGTLC2** | chr22 | + | uc002zwm.3 | 22979652 | 22981152 | + |
| **IGLL1** | chr22 | - | ENST00000458318 | 23909250 | 23915694 | + |
| **TOM1** | chr22 | + | NR_024194 | 35695796 | 35743987 | + |
| **CENPM** | chr22 | - | NR_024355 | 42348190 | 42354946 | + |
| **DRG1** | chr22 | + | uc003akv.1 | 31831222 | 31834552 | + |
| **BID** | chr22 | - | NR_073068 | 18138427 | 18213621 | + |
| **BID** | chr22 | - | NR_073068 | 18138427 | 18213621 | + |
| **ARMCX3** | chrX | + | NR_033670 | 100870107 | 100872991 | - |
| **GK** | chrX | + | ENST00000497961 | 30716323 | 30740049 | + |
| **GK** | chrX | + | ENST00000441146 | 30689751 | 30690283 | + |
| **GK** | chrX | + | ENST00000497961 | 30716323 | 30740049 | + |
| **GK** | chrX | + | ENST00000441146 | 30689751 | 30690283 | + |
| **MAP7D3** | chrX | - | NR_027621 | 135228860 | 135293518 | + |
| **RAB40A** | chrX | - | TCONS_00017355 | 102781044 | 102785739 | - |
| **RBMX** | chrX | - | NR_028476 | 135955605 | 135962939 | - |
| **SAT1** | chrX | + | uc022bty.1 | 23801293 | 23801569 | + |
| **SAT1** | chrX | + | NR_027783 | 23801274 | 23804327 | + |
| **GK** | chrX | + | ENST00000497961 | 30716323 | 30740049 | + |
| **GK** | chrX | + | ENST00000441146 | 30689751 | 30690283 | + |
| **UPRT** | chrX | + | NR_030774 | 74493893 | 74524732 | + |
| **KLF8** | chrX | + | TCONS_00017181 | 56316469 | 56325981 | + |
| **FHL1** | chrX | + | NR_027621 | 135228860 | 135293518 | + |
| **42619** | chrX | - | AK091435 | 118827530 | 118830022 | + |
| **RBMX** | chrX | - | NR_028476 | 135955605 | 135962939 | - |
| **CD24** | chrY | - | ENST00000454875 | 21034388 | 21239004 | - |
| **CD24** | chrY | - | ENST00000331787 | 21094584 | 21239302 | - |

**Table S5. GO clusters of dysregulated adjacent genes of dysregulated lncRNAs.**

| GO_Term | Name | P-Value | FDR |
| --- | --- | --- | --- |
| GO:0007049 | cell cycle | 5.67904E-06 | 0.009715913 |
| GO:0005819 | spindle | 1.30633E-05 | 0.017601754 |
| GO:0000775 | chromosome, centromeric region | 1.37373E-05 | 0.018509773 |
| GO:0051301 | cell division | 1.61798E-05 | 0.027678608 |
| GO:0022402 | cell cycle process | 3.4341E-05 | 0.058738248 |
| GO:0015630 | microtubule cytoskeleton | 5.00567E-05 | 0.067431789 |
| GO:0022403 | cell cycle phase | 5.03908E-05 | 0.086179349 |
| GO:0000279 | M phase | 6.79608E-05 | 0.116211504 |
| GO:0006915 | apoptosis | 9.83479E-05 | 0.168131709 |
| GO:0012501 | programmed cell death | 0.000124454 | 0.212717731 |
| GO:0005876 | spindle microtubule | 0.000147685 | 0.198826997 |
| GO:0006955 | immune response | 0.000152109 | 0.259926818 |
| GO:0044427 | chromosomal part | 0.000203756 | 0.274218139 |
| GO:0005694 | chromosome | 0.000231484 | 0.311481382 |
| GO:0043232 | intracellular non-membrane-bounded organelle | 0.000244008 | 0.328307778 |
| GO:0043228 | non-membrane-bounded organelle | 0.000244008 | 0.328307778 |
| GO:0000278 | mitotic cell cycle | 0.000293428 | 0.500845448 |
| GO:0008219 | cell death | 0.000297064 | 0.507036571 |
| GO:0016265 | death | 0.000329519 | 0.562284803 |
| GO:0007067 | mitosis | 0.00033631 | 0.573842751 |
| GO:0000280 | nuclear division | 0.00033631 | 0.573842751 |
| GO:0000087 | M phase of mitotic cell cycle | 0.00039989 | 0.681978737 |
| GO:0048285 | organelle fission | 0.000492135 | 0.838671548 |
| GO:0000226 | microtubule cytoskeleton organization | 0.000546915 | 0.931614849 |
| GO:0007005 | mitochondrion organization | 0.001384106 | 2.341871235 |
| GO:0042981 | regulation of apoptosis | 0.001680172 | 2.83605642 |
| GO:0005912 | adherens junction | 0.001901476 | 2.532124195 |
| GO:0043067 | regulation of programmed cell death | 0.001942676 | 3.272252426 |
| GO:0007010 | cytoskeleton organization | 0.00194777 | 3.280698912 |
| GO:0005815 | microtubule organizing center | 0.002039795 | 2.713982955 |
| GO:0010941 | regulation of cell death | 0.002044153 | 3.440377539 |
| GO:0006916 | anti-apoptosis | 0.002150693 | 3.616597767 |
| GO:0005814 | centriole | 0.002159625 | 2.87127751 |
| GO:0005925 | focal adhesion | 0.002362534 | 3.137090456 |
| GO:0008637 | apoptotic mitochondrial changes | 0.002788595 | 4.665379244 |
| GO:0005924 | cell-substrate adherens junction | 0.002936136 | 3.884875512 |
| GO:0051297 | centrosome organization | 0.003139499 | 5.237712928 |
| GO:0044450 | microtubule organizing center part | 0.003210861 | 4.241128357 |
| GO:0015031 | protein transport | 0.003229205 | 5.383505441 |
| GO:0044430 | cytoskeletal part | 0.003400396 | 4.486197254 |
| GO:0045184 | establishment of protein localization | 0.003667548 | 6.092882004 |
| GO:0000777 | condensed chromosome kinetochore | 0.003742163 | 4.926635985 |
| GO:0006952 | defense response | 0.003796014 | 6.299829279 |
| GO:0070161 | anchoring junction | 0.003829867 | 5.039356779 |
| GO:0030055 | cell-substrate junction | 0.00398951 | 5.244220754 |
| GO:0031023 | microtubule organizing center organization | 0.004370336 | 7.21977145 |
| GO:0007059 | chromosome segregation | 0.004637472 | 7.64476417 |
| GO:0005856 | cytoskeleton | 0.004692247 | 6.141159122 |
| GO:0002683 | negative regulation of immune system process | 0.005225357 | 8.573594594 |
| GO:0007098 | centrosome cycle | 0.005414577 | 8.870676469 |
| GO:0008104 | protein localization | 0.006131921 | 9.988700545 |
| GO:0000779 | condensed chromosome, centromeric region | 0.006505308 | 8.419088166 |
| GO:0000793 | condensed chromosome | 0.008538599 | 10.9127701 |
| GO:0033363 | secretory granule organization | 0.009545066 | 15.13364159 |
| GO:0007017 | microtubule-based process | 0.009894809 | 15.64490348 |
| GO:0031981 | nuclear lumen | 0.010240012 | 12.95095847 |
| GO:0007051 | spindle organization | 0.010712394 | 16.82877649 |
| GO:0016323 | basolateral plasma membrane | 0.010962473 | 13.80329407 |
| GO:0000776 | kinetochore | 0.012285758 | 15.34445734 |
| GO:0015631 | tubulin binding | 0.012486346 | 16.55997947 |
| GO:0031974 | membrane-enclosed lumen | 0.012602982 | 15.71010093 |
| GO:0042130 | negative regulation of T cell proliferation | 0.014602028 | 22.24989881 |
| GO:0016050 | vesicle organization | 0.017544866 | 26.12844606 |
| GO:0043065 | positive regulation of apoptosis | 0.01913179 | 28.14359943 |
| GO:0043066 | negative regulation of apoptosis | 0.01957953 | 28.70273029 |
| GO:0005813 | centrosome | 0.019674273 | 23.49081157 |
| GO:0051250 | negative regulation of lymphocyte activation | 0.019898081 | 29.09803471 |
| GO:0043068 | positive regulation of programmed cell death | 0.020345356 | 29.64959763 |
| GO:0006917 | induction of apoptosis | 0.020991352 | 30.43908785 |
| GO:0010942 | positive regulation of cell death | 0.021139663 | 30.61916123 |
| GO:0012502 | induction of programmed cell death | 0.021475028 | 31.02473248 |
| GO:0043069 | negative regulation of programmed cell death | 0.021532506 | 31.09401921 |
| GO:0060548 | negative regulation of cell death | 0.022206483 | 31.90158115 |
| GO:0051054 | positive regulation of DNA metabolic process | 0.02243699 | 32.17572555 |
| GO:0032945 | negative regulation of mononuclear cell proliferation | 0.023087282 | 32.94353162 |
| GO:0050672 | negative regulation of lymphocyte proliferation | 0.023087282 | 32.94353162 |
| GO:0070664 | negative regulation of leukocyte proliferation | 0.023087282 | 32.94353162 |
| GO:0006954 | inflammatory response | 0.023419383 | 33.33248337 |
| GO:0050900 | leukocyte migration | 0.023777091 | 33.74904751 |
| GO:0002695 | negative regulation of leukocyte activation | 0.023777091 | 33.74904751 |
| GO:0031109 | microtubule polymerization or depolymerization | 0.024961861 | 35.11132623 |
| GO:0046640 | regulation of alpha-beta T cell proliferation | 0.024961861 | 35.11132623 |
| GO:0005874 | microtubule | 0.026030285 | 29.91152612 |
| GO:0070585 | protein localization in mitochondrion | 0.027087441 | 37.48950435 |
| GO:0006626 | protein targeting to mitochondrion | 0.027087441 | 37.48950435 |
| GO:0016567 | protein ubiquitination | 0.027286377 | 37.70782981 |
| GO:0020037 | heme binding | 0.02900717 | 34.56620737 |
| GO:0050866 | negative regulation of cell activation | 0.029616122 | 40.2117913 |
| GO:0005099 | Ras GTPase activator activity | 0.030592642 | 36.08891119 |
| GO:0000075 | cell cycle checkpoint | 0.030853419 | 41.50277262 |
| GO:0042129 | regulation of T cell proliferation | 0.031196847 | 41.85642074 |
| GO:0050000 | chromosome localization | 0.032768659 | 43.44947066 |
| GO:0051303 | establishment of chromosome localization | 0.032768659 | 43.44947066 |
| GO:0043933 | macromolecular complex subunit organization | 0.032841384 | 43.5221744 |
| GO:0043233 | organelle lumen | 0.032931674 | 36.31591199 |
| GO:0070013 | intracellular organelle lumen | 0.035320132 | 38.40304786 |
| GO:0046906 | tetrapyrrole binding | 0.037921975 | 42.70911027 |
| GO:0031508 | centromeric heterochromatin formation | 0.038070802 | 48.52542246 |
| GO:0070828 | heterochromatin organization | 0.038070802 | 48.52542246 |
| GO:0031507 | heterochromatin formation | 0.038070802 | 48.52542246 |
| GO:0033364 | mast cell secretory granule organization | 0.038070802 | 48.52542246 |
| GO:0046649 | lymphocyte activation | 0.038584132 | 48.99338342 |
| GO:0005794 | Golgi apparatus | 0.040049428 | 42.35013197 |
| GO:0016477 | cell migration | 0.041074726 | 51.2075415 |
| GO:0009132 | nucleoside diphosphate metabolic process | 0.041393414 | 51.4842356 |
| GO:0032446 | protein modification by small protein conjugation | 0.042062342 | 52.06022046 |
| GO:0034621 | cellular macromolecular complex subunit organization | 0.043549025 | 53.3173659 |
| GO:0045321 | leukocyte activation | 0.044442758 | 54.05810124 |
| GO:0005654 | nucleoplasm | 0.044591246 | 45.91910737 |
| GO:0019221 | cytokine-mediated signaling pathway | 0.045608009 | 55.00726212 |
| GO:0009057 | macromolecule catabolic process | 0.045760833 | 55.13036724 |
| GO:0050869 | negative regulation of B cell activation | 0.045989512 | 55.313984 |
| GO:0016574 | histone ubiquitination | 0.045989512 | 55.313984 |
| GO:0050868 | negative regulation of T cell activation | 0.046506494 | 55.726488 |
| GO:0030098 | lymphocyte differentiation | 0.048424499 | 57.22576658 |
| GO:0042127 | regulation of cell proliferation | 0.050079506 | 58.48091262 |
